# Real‐world treatment trajectories preceding GLP‐1 receptor agonist initiation in type 2 diabetes: A descriptive UK population‐based cohort study on adherence to national clinical guidelines

**DOI:** 10.1111/dom.70548

**Published:** 2026-02-25

**Authors:** Franziska S. Ulrich, Morten Frost Nielsen, Nicola Napoli, Andrea M. Burden

**Affiliations:** ^1^ Institute of Pharmaceutical Sciences ETH Zurich Zurich Switzerland; ^2^ Department of Endocrinology Odense University Hospital Odense Denmark; ^3^ Steno Diabetes Centre Odense Odense University Hospital Odense Denmark; ^4^ Unit of Endocrinology and Diabetes Campus Bio‐Medico University of Rome Rome Italy; ^5^ Unit of Metabolic Bone and Thyroid Disorders Fondazione Policlinico Universitario Campus Bio‐Medico University of Rome Rome Italy; ^6^ Division of Bone and Mineral Diseases Washington University St Louis Missouri USA; ^7^ Division of Clinical Immunology and Rheumatology, Heersink School of Medicine University of Alabama at Birmingham Birmingham Alabama USA

**Keywords:** cardiovascular disease, clinical guidance, drug utilisation, GLP‐1 receptor agonist, glucose‐lowering therapy, obesity, primary care, real‐world evidence, type 2 diabetes, United Kingdom

## Abstract

**Aims:**

To investigate glucose‐lowering treatment trajectories preceding glucagon‐like peptide‐1 receptor agonist (GLP‐1RA) initiation in UK primary care, while assessing alignment with contemporary UK clinical guidelines, considering calendar time, cardiovascular disease (CVD) history, and obesity status.

**Materials and Methods:**

Using the IQVIA Medical Research Data (IMRD) incorporating data from THIN, a Cegedim Database, we included adults with type 2 diabetes initiating GLP‐1RAs in UK primary care between 01 January 2007 and 30 June 2023. We described treatment trajectories from the first glucose‐lowering therapy to GLP‐1RA initiation, stratified by calendar time (GLP‐1RA initiation pre‐ or post‐01 January 2018, when guidelines started recommending sodium‐glucose co‐transporter‐2 inhibitors [SGLT‐2is] for those at high risk or established CVD), CVD history, and body mass index (BMI) ≥35 kg/m^2^.

**Results:**

We included 29 780 GLP‐1RA initiators, with 62.2% (*n* = 18 517) initiating pre‐2018 and 37.8% (*n* = 11 263) post‐2018. Consistent over calendar time, most GLP‐1RA initiators (63.5%) received their first GLP‐1RA as fourth‐line (35.4%) or later‐line therapy (28.1%), with fewer initiating GLP‐1RAs as first‐ (0.8%), second‐ (10.5%), or third‐line (25.2%) treatments. After 2018, 50.8% of individuals initiating GLP‐1RA therapy used SGLT‐2is concomitantly, regardless of CVD status (47.8% with established CVD vs. 51.5% without). Individuals with BMI ≥35 kg/m^2^ initiated GLP‐1RA therapy earlier compared to those with BMI <35 kg/m^2^ (46.3% vs. 29.1% as first‐, second‐, or third‐line treatment).

**Conclusions:**

GLP‐1RAs were predominantly initiated following ≥3 glucose‐lowering agents, consistent with contemporary UK guidance (NG28). Post‐2018, most GLP‐1RA initiators received SGLT‐2is concomitantly. However, CVD history did not influence prescribing patterns, underscoring missed opportunities to optimise the prevention of cardiovascular events and slow the progression of CVD.

## 1 INTRODUCTION

Globally, more than half a billion adults are currently living with type 2 diabetes, a number projected to reach more than 750 million by 2050.^1^ Type 2 diabetes is a major driver of microvascular (e.g., nephropathy) and macrovascular complications such as cardiovascular disease (CVD), largely due to the co‐occurrence of shared cardio‐renal‐metabolic risk factors such as adiposity, inflammation, hypertension, and dyslipidaemia.^2^, ^3^, ^4^ Given the complex and progressive nature of type 2 diabetes, effective management typically requires a multifactorial approach combining lifestyle and behavioural modifications with pharmacotherapy to support optimal glycaemic control and reduce the risk of cardio‐renal‐metabolic complications.^2^, ^3^

Accordingly, the American Diabetes Association (ADA), the European Association for the Study of Diabetes (EASD), and the European Society of Cardiology (ESC) recommend prioritising glucose‐lowering therapies with proven cardio‐renal benefits, specifically glucagon‐like peptide‐1 receptor agonists (GLP‐1RAs) and sodium‐glucose co‐transporter‐2 inhibitors (SGLT‐2is), particularly for individuals with type 2 diabetes who have, or are at high risk of, CVD, heart failure and/or chronic kidney disease (CKD).^5^, ^6^, ^7^ In contrast, the United Kingdom (UK) National Institute for Health and Care Excellence (NICE) guideline NG28 only recommends GLP‐1RAs if triple oral glucose‐lowering therapy failed or is inadequate among individuals with type 2 diabetes who either (1) have a body mass index (BMI) ≥35 kg/m^2^ and obesity‐related complications or who (2) have a BMI <35 kg/m^2^ and insulin therapy would cause occupational implications.^8^ Additionally, while ADA‐EASD guidelines endorse the initiation of GLP‐1RAs and SGLT‐2is irrespective of glycaemic control and prior metformin use in individuals with cardio‐renal risk,^5^, ^6^, ^7^ the NICE guideline NG28 recommends initiating SGLT‐2is only after metformin for those with type 2 diabetes and established CVD, elevated CVD risk and/or CKD.^8^

Consequently, patients with type 2 diabetes managed in the UK typically experience a longer treatment pathway to both SGLT‐2i and, in particular, GLP‐1RA therapy,^8^ compared to countries adhering to ADA‐EASD guidelines.^5^, ^6^, ^7^, ^9^ Nevertheless, while previous studies have shown that prescribing practices in the UK typically follow the NICE guidance,^9^, ^10^ the extent to which clinicians adhere to these recommendations when initiating GLP‐1RA therapy remains unclear.

Therefore, we conducted a population‐based study using UK electronic medical records (EMRs) to descriptively characterise glucose‐lowering treatment pathways leading to GLP‐1RA initiation between 2007 and 2023 in primary care and to assess subsequent treatment modifications. To further inform person‐centred treatment decision‐making and assess alignment with evolving UK clinical guidelines (NG28, published in 2015 and last updated in 2022),^8^ we assessed heterogeneity by calendar time, CVD status, BMI, and sex assigned at birth.

## 2 MATERIALS AND METHODS

### 2.1 Data source and cohort identification

This UK population‐based cohort study used the IQVIA Medical Research Data (IMRD) incorporating data from THIN, a Cegedim Database. Comparable in scope and structure to the widely used Clinical Practice Research Datalink (CPRD) GOLD, the IMRD contains EMRs from general practitioner (GP) practices across the UK, including more than 25 million individuals (as of November 2022).^11^, ^12^, ^13^ The IMRD is broadly representative of the UK general population and provides longitudinal information on demographics, medical diagnoses and procedures, laboratory tests (e.g., glycated haemoglobin [HbA1c]), medication prescriptions, and lifestyle factors (e.g., smoking).^11^, ^12^, ^14^

Within the IMRD, we assembled a cohort of adults (≥18 years) who initiated GLP‐1RA therapy (Table S1 for exposure definition) between 01 January 2007 (i.e., based on the market authorisation of the first GLP‐1RA)^15^ and the end of data collection (i.e., 30 June 2023). Cohort entry was defined as the date of the first prescription of a GLP‐1RA within this period. To be included, individuals were required to have ≥1 year of medical history with the current GP practice and a diagnosis of type 2 diabetes before or at cohort entry. Excluded were individuals with a history of type 1 diabetes, gestational diabetes, or polycystic ovary syndrome (because glucose‐lowering agents can be used for non‐type 2 diabetes indications in patients with this condition) before or at cohort entry, as well as those who received insulin as first‐line glucose‐lowering therapy.^9^ Furthermore, individuals were excluded if they initiated more than one GLP‐1RA agent or combined with insulin (see Table S1). Eligible individuals were followed from GLP‐1RA initiation until the earliest occurrence of death, transfer out of the GP practice, end of data collection, or discontinuation of GLP‐1RA therapy (i.e., having a gap of more than 90 days between consecutive GLP‐1RA prescriptions, regardless of switching between agents).^9^, ^16^

### 2.2 Exposure definition

We identified prescriptions issued by GPs for glucose‐lowering therapies (Table S2) recorded in the IMRD between 01 January 1995 and the end of data collection (30 June 2023). Glucose‐lowering therapies were classified as GLP‐1RAs, metformin, sulfonylureas, SGLT‐2is, dipeptidyl peptidase‐4 inhibitors (DPP‐4is), thiazolidinediones, other oral glucose‐lowering drugs and insulin, while combination product prescriptions were separated into their individual components (Table S2). First‐line treatment was defined as the first non‐insulin glucose‐lowering therapy prescribed, while second‐line to eighth‐line treatments were defined as the initiation of any new glucose‐lowering therapy (including insulin) not used in a previous treatment line.

### 2.3 Cohort characteristics

To describe eligible individuals at GLP‐1RA initiation, we assessed their characteristics during the year before and at cohort entry, including demographics (e.g., age), lifestyle factors (e.g., smoking status), clinical measurements (e.g., BMI and HbA1c), use of other medications (e.g., SGLT‐2is), as well as lifetime history of comorbidities (e.g., CKD and sleep apnoea).

### 2.4 Prescribing trajectories of glucose‐lowering therapies leading to GLP‐1RA initiation

To analyse treatment trajectories leading to GLP‐1RA initiation, we identified the number of individuals who initiated a new glucose‐lowering therapy at each treatment line available (i.e., first to eighth) and visualised utilisation patterns using Sankey diagrams.^17^

### 2.5 Changes in use of glucose‐lowering therapies upon GLP‐1RA initiation

To assess treatment changes around GLP‐1RA initiation (Day 0), we examined the use of glucose‐lowering therapies in four consecutive 180‐day intervals, covering the period from 1 year before to 1 year after GLP‐1RA initiation (*T*_−1_ [−361, −181], *T*_0_ [−180, 0], *T*_+1_ [1, 181], and *T*_+2_ [182, 362] days). All glucose‐lowering agents prescribed within a given 180‐day interval were considered to be used concomitantly.^3^

### 2.6 Subgroup analyses

Given the availability of newer glucose‐lowering agents and changing NICE guidance, we conducted our analyses stratified by calendar time (based on the date of GLP‐1RA initiation) using 01 January 2018 as the cutoff. This date marks when type 2 diabetes guidelines started to recommend cardiovascular risk‐based therapy and expanded second‐line options, specifically prioritising SGLT‐2is following metformin for individuals with established or high risk of CVD.^8^, ^9^ As NICE guidance on GLP‐1RA use remained largely unchanged over the study period, this stratification was intended to capture changes in prescribing patterns preceding GLP‐1RA initiation rather than changes in GLP‐1RA use itself.^8^, ^9^ To further assess whether UK prescribing patterns of GLP‐1RAs align with contemporary NICE guidelines (NG28), we additionally stratified post‐2018 analyses by (1) the presence of a CVD history at cohort entry and (2) BMI categories <35 and ≥35 kg/m^2^ for individuals with a BMI measurement recorded within the year before or at cohort entry. Individuals without a recent BMI measurement were assigned to a dedicated ‘missing’ category. CVD history was defined as a recorded diagnosis of myocardial infarction, stable or unstable angina, coronary atherosclerosis, transient ischaemic attack or stroke, coronary procedures, or heart failure prior to or at cohort entry. Furthermore, we examined differences in GLP‐1RA initiation patterns by sex assigned at birth.

All analyses were conducted using the statistical software R (version 4.2.2; R Core Team, 2022) and SAS (version 9.4; SAS Institute Inc., 2016).

## 3 RESULTS

We identified 29 780 eligible adults with type 2 diabetes who initiated GLP‐1RA therapy during 2007–2023 within UK primary care (Figure S1).

### 3.1 Characteristics of GLP‐1RA therapy initiators

The characteristics of the cohort at GLP‐1RA initiation are summarised in Table 1, stratified by calendar period of initiation (i.e., before 2018 vs. in 2018 and later). Among all initiators, 44.2% were female, with a median age of 59.0 years (interquartile range [IQR] 51.5–66.3), HbA1c of 8.2% (IQR 7.7–8.6), and type 2 diabetes duration of 6.4 years (IQR 3.4–10.3). Moreover, the majority of individuals (*n* = 20 185; 85.9%) with a recorded BMI measurement (*n* = 23 494) met the World Health Organization criteria for obesity (i.e., BMI ≥30 kg/m^2^).^18^ Having a history of obesity‐ and type 2 diabetes‐related comorbidities was common, including CVD (18.3%), hypertension (66.1%), depression (38.0%), and CKD (15.3%). Prior to initiating GLP‐1RA therapy, most individuals (69.0%) were already prescribed three or more glucose‐lowering therapies.

**TABLE 1:** Characteristics of individuals with type 2 diabetes initiating glucagon‐like peptide‐1 (GLP‐1) receptor agonist therapy between 2007 and 2023, stratified by initiation before 2018 vs. in 2018 and later.

Characteristics	Overall (*n* = 29 780)	Initiation pre‐2018 (*n* = 18 517)	Initiation post‐2018 (*n* = 11 263)
Age at initiation (years)	59.0 [51.5, 66.3]	58.0 [50.6, 65.0]	60.8 [53.1, 68.6]
Female sex at birth	13 157 (44.2%)	8123 (43.9%)	5034 (44.7%)
Type 2 diabetes duration^a^ (years)	6.4 [3.4, 10.3]	6.3 [3.5, 9.7]	6.7 [3.2, 11.5]
GLP‐1 receptor agonist agent initiated			
Semaglutide	5084 (17.1%)	<7	5084 (45.1%)
Dulaglutide	5665 (19.0%)	1003 (5.4%)	4662 (41.4%)
Liraglutide	10 017 (33.6%)	8783 (47.4%)	1234 (11.0%)
Exenatide	7912 (26.6%)	7671 (41.4%)	241 (2.1%)
Lixisenatide	1102 (3.7%)	1060 (5.7%)	42 (0.4%)
Previous use of glucose‐lowering therapies^b^			
Metformin	29 289 (98.4%)	18 357 (99.1%)	10 932 (97.1%)
Sulfonylureas	20 267 (68.1%)	13 981 (75.5%)	6286 (55.8%)
SGLT‐2 inhibitors	8093 (27.2%)	1266 (6.8%)	6827 (60.6%)
DPP‐4 inhibitors	14 823 (49.8%)	8367 (45.2%)	6456 (57.3%)
Thiazolidinediones	10 060 (33.8%)	8335 (45.0%)	1725 (15.3%)
Insulin	5345 (17.9%)	3897 (21.0%)	1448 (12.9%)
Other glucose‐lowering therapies	1386 (4.7%)	1254 (6.8%)	132 (1.2%)
Number of glucose‐lowering therapies ≥3	20 546 (69.0%)	12 967 (70.0%)	7579 (67.3%)
Concurrent use of glucose‐lowering therapies^c^			
Metformin	26 197 (88.0%)	16 662 (90.0%)	9535 (84.7%)
Sulfonylureas	15 053 (50.5%)	10 704 (57.8%)	4349 (38.6%)
SGLT‐2 inhibitors	6828 (22.9%)	1105 (6.0%)	5723 (50.8%)
DPP‐4 inhibitors	12 019 (40.4%)	7117 (38.4%)	4902 (43.5%)
Thiazolidinediones	4411 (14.8%)	3911 (21.1%)	500 (4.4%)
Insulin	4878 (16.4%)	3574 (19.3%)	1304 (11.6%)
Other glucose‐lowering therapies	354 (1.2%)	327 (1.8%)	27 (0.2%)
Number of glucose‐lowering therapies ≥3	13 190 (44.3%)	8051 (43.5%)	5139 (45.6%)
Comorbidities^d^			
Cardiovascular disease	5459 (18.3%)	3356 (18.1%)	2103 (18.7%)
Myocardial infarction	1759 (5.9%)	988 (5.3%)	771 (6.8%)
Stroke	953 (3.2%)	532 (2.9%)	421 (3.7%)
Heart failure	892 (3.0%)	487 (2.6%)	405 (3.6%)
Hypertension	19 699 (66.1%)	12 525 (67.6%)	7174 (63.7%)
Dyslipidaemia	6583 (22.1%)	4394 (23.7%)	2189 (19.4%)
Sleep apnoea	1399 (4.7%)	851 (4.6%)	548 (4.9%)
Asthma	6536 (21.9%)	3996 (21.6%)	2540 (22.6%)
Chronic obstructive pulmonary disease	7522 (25.3%)	4631 (25.0%)	2891 (25.7%)
Chronic kidney disease	4569 (15.3%)	2795 (15.1%)	1774 (15.8%)
Osteoarthritis	6558 (22.0%)	3901 (21.1%)	2657 (23.6%)
Depression	11 310 (38.0%)	6690 (36.1%)	4620 (41.0%)
Cancer	2022 (6.8%)	1057 (5.7%)	965 (8.6%)
Comedications^e^			
Statins	24 199 (81.3%)	15 466 (83.5%)	8733 (77.5%)
Angiotensin converting enzyme inhibitors	15 216 (51.1%)	10 014 (54.1%)	5202 (46.2%)
Angiotensin II receptor blockers	6124 (20.6%)	4040 (21.8%)	2084 (18.5%)
Diuretics	9659 (32.4%)	6634 (35.8%)	3025 (26.9%)
Calcium channel blockers	1040 (3.5%)	780 (4.2%)	260 (2.3%)
Beta‐blockers	7869 (26.4%)	4727 (25.5%)	3142 (27.9%)
Acetylsalicylic acid	9964 (33.5%)	7598 (41.0%)	2366 (21.0%)
Other antiplatelets	2084 (7.0%)	1170 (6.3%)	914 (8.1%)
Anticoagulants	2047 (6.9%)	1039 (5.6%)	1008 (8.9%)
Laboratory and vital sign measurements^f^			
BMI (kg/m^2^)	35.4 [31.8, 39.3]	36.1 [32.7, 40.0]	34.0 [30.5, 38.0]
BMI <30 kg/m^2^	3309 (11.1%)	1478 (8.0%)	1831 (16.3%)
BMI 30 to <35 kg/m^2^	7766 (26.1%)	4684 (25.3%)	3082 (27.4%)
BMI ≥35 kg/m^2^	12 419 (41.7%)	8765 (47.3%)	3654 (32.4%)
BMI missing	6286 (21.1%)	3590 (19.4%)	2696 (23.9%)
HbA1c (%)	8.2 [7.7, 8.6]	8.2 [7.7, 8.6]	8.2 [7.6, 8.6]
eGFR (mL/min per 1.73 m^2^)	98.9 [83.8, 108.2]	99.4 [84.7, 108.4]	98.2 [82.0, 107.7]
Systolic blood pressure (mmHg)	134.0 [124.0, 141.0]	134.0 [125.0, 142.0]	133.0 [124.0, 140.0]
Diastolic blood pressure (mmHg)	80.0 [72.0, 84.0]	80.0 [72.0, 84.0]	80.0 [72.0, 84.0]
Serum cholesterol (mmol/L)	4.2 [3.6, 4.9]	4.2 [3.6, 4.8]	4.3 [3.6, 5.1]
Smoking^g^			
Markers of current smoking	4386 (14.7%)	2804 (15.1%)	1582 (14.0%)

*Note*: Data presented in *n* (%) or median [IQR]. Counts <7 are suppressed to prevent person identification.

Abbreviations: BMI, body mass index; DPP‐4, dipeptidyl peptidase‐4; eGFR, estimated glomerular filtration rate; HbA1c, glycated haemoglobin; SGLT‐2, sodium‐glucose co‐transporter‐2.

^a^ Time (years) between the first non‐insulin glucose‐lowering therapy initiated and GLP‐1 receptor agonist initiation.

^b^ Defined as having at least one prescription for a glucose‐lowering therapy specified in Table S2 before GLP‐1 receptor agonist initiation.

^c^ Defined as having at least one prescription for a glucose‐lowering therapy specified in Table S2 within the 180 days before or at GLP‐1 receptor agonist initiation.

^d^ Diagnosis recorded before or at GLP‐1 receptor agonist initiation.

^e^ Defined as having at least one prescription within the 365 days before or at GLP‐1 receptor agonist initiation.

^f^ Most recent measurement recorded within the 365 days before or at GLP‐1 receptor agonist initiation. The proportions of missing values were 2.1%–7.6% for blood pressure (systolic and diastolic), 3.0%–3.1% for eGFR, 8.7%–11.5% for serum cholesterol and 38.4%–53.6% for HbA1c.

^g^ Most recent record before or at GLP‐1 receptor agonist initiation.

If the first GLP‐1RA was prescribed before 2018 (*n* = 18 517), the initiation of older GLP‐1RA agents was more common, such as liraglutide (47.4%) or exenatide (41.4%), while co‐prescribed glucose‐lowering therapies were most often metformin (90.0%), sulfonylureas (57.8%), DPP‐4is (38.4%), and thiazolidinediones (21.1%), rather than SGLT‐2is (6.0%).

After 2018 (*n* = 11 263), the most frequently initiated GLP‐1RA agents were semaglutide (45.1%), dulaglutide (41.4%), or liraglutide (11.0%), while the most commonly co‐prescribed glucose‐lowering therapies were metformin (84.7%), SGLT‐2is (50.8%), DPP‐4is (43.5%), and sulfonylureas (38.6%).

### 3.2 Patterns of GLP‐1RA therapy initiation across glucose‐lowering treatment lines

**FIGURE 1:**
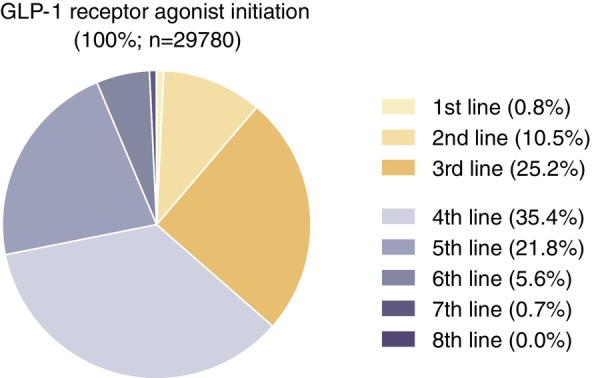
Distribution of glucagon‐like peptide‐1 (GLP‐1) receptor agonist initiation between 2007 and 2023 across glucose‐lowering treatment lines. First‐line treatment was defined as the first non‐insulin glucose‐lowering therapy prescribed, while second‐line to eighth‐line treatments were defined as the initiation of any new glucose‐lowering therapy (including insulin), not used in a previous treatment line. The figure was created using GraphPad Prism version 10.2.0 [335].

Figure 1 presents the distribution of GLP‐1RA initiation at different lines of glucose‐lowering therapy. Overall, the majority of GLP‐1RA initiators (63.5%) received their first GLP‐1RA within UK primary care as a fourth‐line (35.4%) or later therapy line (i.e., 28.1% in fifth to eighth‐line). Only 0.8%, 10.5%, and 25.2% of individuals initiated GLP‐1RA therapy as first‐, second‐, and third‐line treatments, respectively (Figure 1 and Table S3). The patient characteristics stratified by treatment lines were consistent across calendar time strata (Tables S3–S5). Individuals initiating GLP‐1RAs in earlier treatment lines (e.g., first‐ to third‐line therapy) were more likely to be younger, have higher BMI, and a history of depression, asthma, or chronic obstructive pulmonary disease. In contrast, later‐line GLP‐1RA initiation was associated with longer diabetes duration and a greater burden of cardiometabolic comorbidities, including CVD, CKD, osteoarthritis, and dyslipidaemia, consistent with progressive disease and stepwise treatment intensification.

### 3.3 Glucose‐lowering therapy pathways preceding GLP‐1RA therapy initiation

**FIGURE 2:**
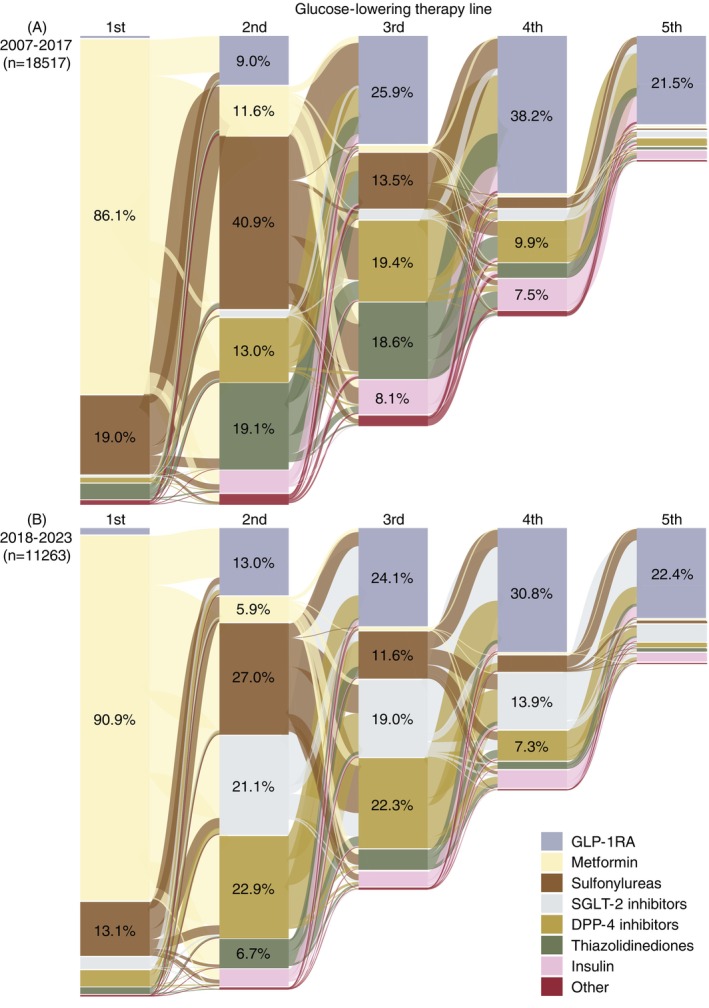
Sankey diagrams showing the trajectories of glucose‐lowering therapies from the first prescription to the initiation of glucagon‐like peptide‐1 receptor agonist (GLP‐1RA) therapy between (A) 2007–2017 and (B) 2018–2023, stratified by treatment line. First‐line treatment was defined as the first non‐insulin glucose‐lowering therapy prescribed, while second‐line to eighth‐line treatments were defined as the initiation of any new glucose‐lowering therapy (including insulin), not used in a previous treatment line. For clarity, trajectories are displayed up to the fifth treatment line; non‐GLP‐1RA therapies shown in the fifth line represent individuals who initiated a GLP‐1RA at the sixth‐line or later. Percentages of individuals initiating a given therapy at each treatment line are displayed within columns when >5%. Complete numerical results for all treatment lines are reported in Table S6, while the diagrams provide a visual summary of treatment sequencing across lines. Sankey diagrams were generated using the R package networkD3.^17^ DPP‐4, dipeptidyl peptidase‐4; SGLT‐2, sodium‐glucose co‐transporter‐2.

The Sankey diagrams shown in Figure 2A,B (calendar period stratifications) illustrate the real‐world transitions between glucose‐lowering therapies from the first prescription up to the initiation of GLP‐1RA therapy. The corresponding diagram for the overall cohort is shown in Figure S2, while the underlying numbers for all Sankey diagrams are provided in Table S6.

Across calendar time, metformin was consistently the predominant first‐line treatment initiated in over 85% of individuals. Before 2018 (Figure 2A), treatment pathways typically included sulfonylureas (40.9%) or thiazolidinediones (19.1%) as second‐line treatments. Transitions to GLP‐1RAs commonly occurred following sulfonylureas, thiazolidinediones, DPP‐4is and/or insulin. After 2018 (Figure 2B), treatment trajectories shifted with second‐line treatments increasingly including SGLT‐2is (21.1%) and DPP‐4is (22.9%) compared to 1.1% and 13.0% before 2018. This trend was also observed across third‐ and fourth‐treatment lines, with SGLT‐2is initiated in 19.0% and 13.9% and DPP‐4is in 22.3% and 7.3% of individuals who started their first GLP‐1RA after 2018, respectively.

### 3.4 Treatment modifications upon GLP‐1RA initiation

**FIGURE 3:**
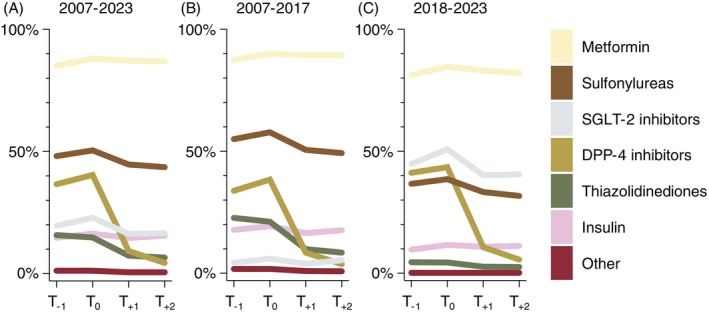
Prescribing patterns of glucose‐lowering therapies (GLT) during the 1‐year before and after glucagon‐like peptide‐1 receptor agonist (GLP‐1RA) initiation, (A) overall, and stratified by initiation (B) before 2018, and (C) in 2018 or later. The use of GLT was assessed in four time intervals (days) relative to GLP‐1RA initiation at Day 0: (1) *T*_−1_ (−361 to −181 days), (2) *T*_0_ (−180 to 0 days), (3) *T*_+1_ (1 to 181 days), and (4) *T*_+2_ (182 to 362 days), while all agents prescribed in an interval were considered as concomitantly used. The corresponding numbers of individuals prescribed specific GLT for each interval are displayed in Table S7. DPP‐4, dipeptidyl peptidase‐4; SGLT‐2, sodium‐glucose co‐transporter 2. Graphs were created using GraphPad Prism (version 10.2.0 [335]).

Figure 3A–C depict the patterns of glucose‐lowering therapy use during the year before and after GLP‐1RA initiation, both overall and stratified by calendar period (before 2018 vs. 2018 and later). Consistent across calendar periods, prescribing of metformin and insulin remained relatively stable before and after GLP‐1RA initiation, whereas the use of sulfonylureas, SGLT‐2is and thiazolidinediones declined slightly, and DPP‐4i prescribing decreased sharply post‐initiation (Figure 3A–C and Table S7). Within the pre‐2018 cohort (Figure 3B), metformin (89.4%) and sulfonylureas (50.6%) stayed the most commonly co‐prescribed agents in the period after GLP‐1RA initiation, while in the post‐2018 cohort (Figure 3C), SGLT‐2is (40.2%) remained the second most frequently co‐prescribed class after metformin post‐initiation (83.1%).

### 3.5 Prescribing patterns after 2018 by CVD history, BMI, and sex assigned at birth

In the post‐2018 cohort of GLP‐1RA initiators (*n* = 11 263), 18.7% (*n* = 2103) had established CVD (Table 1 and Table S8). Individuals with established CVD had a longer duration of type 2 diabetes at the time of GLP‐1RA initiation than those without (median 7.8 [IQR 3.9–13.2] vs. 6.5 [IQR 3.1–11.1] years). Yet, concomitant use of SGLT‐2is at GLP‐1RA initiation was similar among individuals with CVD (47.8%) and those without CVD (51.5%). Likewise, early‐line GLP‐1RA initiation (first‐ to third‐line therapy) was not more common in the CVD group (34.5% vs. 39.4%) (Tables S8–S10 and Figure S3A,B).

Restricting the post‐2018 cohort to individuals with a BMI measurement recorded (*n* = 8567) and stratifying by BMI category, semaglutide and liraglutide, the two GLP‐1RAs additionally recommended for weight management by UK's NICE guidance,^19^ were not preferentially initiated among those with a BMI ≥35 kg/m^2^ (42.7%; *n* = 3654) compared to those with a BMI <35 kg/m^2^ (57.3%; *n* = 4913; Table S11). However, individuals with a BMI ≥35 kg/m^2^ more frequently initiated GLP‐1RAs in earlier stages of treatment, with 46.3% receiving them as first‐, second‐ or third‐line therapy compared to 29.1% among those with a BMI <35 kg/m^2^ and thus were less likely to be prescribed ≥3 concomitant glucose‐lowering therapies at initiation (38.0% vs. 55.1%; Tables S12 and S13 and Figure S4A,B).

When stratifying the post‐2018 cohort by sex assigned at birth (44.7% females; *n* = 5034), the trajectories and utilisation of glucose‐lowering treatments were similar across sexes (see Tables S14–S16 and Figures S5A,B).

## 4 DISCUSSION

This descriptive UK population‐based study provides a comprehensive analysis of the road towards GLP‐1RA initiation and subsequent treatment modifications among adults with type 2 diabetes managed in UK primary care during 2007–2023. Across this period, prescribing patterns were largely consistent with contemporary UK guidelines (NG28, last updated in 2022), which recommend GLP‐1RA therapy if prior triple therapy with metformin and two other oral glucose‐lowering drugs was ineffective, not tolerated, or contraindicated.^8^ However, prescribing patterns also revealed deviations from UK guidance‐recommended care and identified opportunities to optimise treatment delivery, particularly among individuals with established CVD and/or obesity.

Across all calendar periods, the majority of individuals (69.0%) initiated GLP‐1RA therapy after receiving prescriptions for at least three different glucose‐lowering therapies. Initiation most commonly occurred as the fourth‐line (35.4%) or fifth‐line (21.8%) treatment option, and rarely as first‐line (0.8%), second‐line (10.5%), or third‐line (25.2%) therapies. These observations align with previous research demonstrating that GLP‐1RAs are infrequently used as second‐ or third‐line treatments in UK clinical practice.^9^

In contrast to contemporary UK guidance (NG28),^8^ ADA‐EASD and ESC guidelines emphasise the importance of initiating GLP‐1RAs and/or SGLT‐2is early, irrespective of glycaemic control and prior metformin use, to improve cardiovascular risk factors (e.g., blood pressure, lipid profile), to prevent and delay cardio‐renal complications (e.g., cardiovascular events, heart failure, and CKD), and other obesity‐related conditions (e.g., nonalcoholic steatohepatitis and sleep apnoea), particularly in individuals with established or high risk of CVD, heart failure, CKD or overweight/obesity.^5^, ^6^, ^7^, ^20^, ^21^, ^22^, ^23^, ^24^ We observed, however, early GLP‐1RA initiation (first‐ to third‐line) was less frequent among individuals with established CVD (34.5%) compared to those without (39.4%). Moreover, while we observed a marked increase in SGLT‐2i prescribing prior to GLP‐1RA therapy initiation in the UK after 2018, which is presumably driven by evidence from large clinical trials and clinical guidelines recommending SGLT‐2is following metformin for individuals at CVD risk,^8^, ^25^, ^26^, ^27^ we did not observe preferential co‐prescribing of SGLT‐2is among patients with a history of CVD. Abrahami et al.,^9^ analysing UK primary care data up to June 2021, suggested that lack of guideline penetration could explain this trend—namely, the prescribing patterns observed in clinical practice had not yet caught up with most recent recommendations. However, our analysis extended through June 2023, covering a period during which awareness and uptake of guidelines would be expected to have improved, suggesting that delayed adoption alone is no longer an adequate explanation.

Furthermore, sulfonylureas were still co‐prescribed in 38.6% of individuals at the time of GLP‐1RA initiation after 2018, including those with established CVD. This is concerning given the potential for sulfonylurea‐induced hypoglycaemia to exacerbate cardiovascular risk.^28^, ^29^ While ADA‐EASD guidelines have advised limiting sulfonylurea use due to their lack of cardio‐renal benefit and association with hypoglycaemia and weight gain, their continued use in UK primary care is plausibly driven by cost and accessibility.^5^, ^6^

Similarly, in spite of the recommendations from NICE guidance on overweight and obesity management advocating the use of semaglutide and liraglutide in individuals with BMI ≥35 kg/m^2^ for weight loss, irrespective of diabetes status,^19^ these agents were not preferentially prescribed to this cohort. Nevertheless, we did observe that early‐line use (first‐ to third‐line) of GLP‐1RAs was more common among individuals with a BMI ≥35 kg/m^2^ (46.3%) compared to those with a BMI <35 kg/m^2^ (29.1%). This indicates that GLP‐1RAs were initiated earlier than recommended by contemporary UK guidelines (NG28) in type 2 diabetes, particularly among individuals with higher BMI, likely reflecting considerations on benefits of earlier use in the context of obesity and related comorbidities.^8^ Given that weight loss is a key component of type 2 diabetes management, and that over half (52.9%) of individuals in the overall study cohort with a recorded BMI had a BMI ≥35 kg/m^2^, this suggests that incorporating a stronger emphasis on obesity management within the context of type 2 diabetes in UK guidelines could potentially enhance clinical outcomes.^8^ In contrast, EASD‐ADA guidelines already recommend semaglutide or tirzepatide, considered as high‐efficacy agents for weight loss, as part of the treatment strategy to achieve and maintain both glycaemic control and weight management targets.^5^, ^6^

Taken together, our findings underscore the potential to optimise treatment pathways in type 2 diabetes, particularly among individuals with coexisting obesity and those at high cardiovascular risk, where important gaps in care persist. While ADA‐EASD guidelines recommend early use of GLP‐1RAs and SGLT‐2is, irrespective of glycaemic control, such strategies have not been fully integrated into UK clinical guidance. This misalignment points to the importance of translating emerging clinical evidence into national treatment guidelines and re‐evaluating structural barriers (e.g., cost‐effectiveness thresholds) that may limit patient access to clinically appropriate therapies.

In addition to the clinical implications, the patterns identified in our study highlight how clinical frameworks and guidelines can alter drug utilisation, generating challenges for conducting comparative safety or effectiveness studies, where selecting an appropriate active comparator (i.e., a drug prescribed to individuals with similar characteristics and clinical indications) is essential to mitigate confounding and bias.^9^, ^30^, ^31^, ^32^ For example, Abrahami et al.^9^ using US Optum Clinformatics data reported GLP‐1RAs and SGLT‐2is were initiated as second‐line therapies among 12.6% and 15.0% of metformin users, respectively. However, in our UK‐based study, we observed that GLP‐1RAs were predominantly initiated after receiving ≥3 different glucose‐lowering agents, including SGLT‐2is (60.6%), DPP‐4is (57.3%), and sulfonylureas (55.8%) in the post‐2018 cohort, all recommended as second‐line options by contemporary NICE guidelines (NG28).^8^ Our findings therefore indicate that GLP‐1RAs are typically reserved for a distinct subgroup of individuals in the UK—those with more advanced type 2 diabetes, prior exposure to multiple glucose‐lowering therapies, higher BMI and obesity‐related complications—unlike sulfonylureas, SGLT‐2is, or DPP‐4is. Consequently, employing an incident new‐user design in comparative studies may introduce biases such as confounding by disease severity.^30^, ^32^, ^33^ Thus, alternative designs, such as a prevalent new‐user design, incorporating careful matching based on prior disease and treatment history, may be better suited to provide insights into the safety and effectiveness of GLP‐1RAs within the UK clinical setting, while capturing dynamic treatment patterns of type 2 diabetes.^32^, ^34^

This study has several strengths. Leveraging a longitudinal UK primary care database representative of the general UK population enabled a robust assessment of dynamic prescribing patterns of glucose‐lowering therapies over time—offering a more nuanced perspective than prior cross‐sectional analyses. Our treatment trajectory approach provides valuable insights into the real‐world clinical management of type 2 diabetes, particularly by highlighting transitions between therapies and clinical decision‐making for relevant patient subgroups.

However, limitations should be acknowledged. First, exposure and follow‐up misclassification are inherent to analyses of UK primary care EMRs. GLP‐1RA prescriptions issued in secondary care, including those prescribed for weight‐management indications, may have led to slight underestimation of GLP‐1RA utilisation.^19^ In addition, follow‐up ended when individuals transferred out of their GP practice; therefore, prescriptions recorded thereafter were not observed, which may have resulted in underestimation of treatment persistence and post‐GLP‐1RA initiation treatment modifications. Nevertheless, as long‐term management of type 2 diabetes in the UK is predominantly delivered and recorded in primary care, these limitations are unlikely to materially affect the interpretation of our findings. Second, although all individuals were required to have at least 1 year of continuous registration with their current GP practice prior to GLP‐1RA initiation, treatment history before registration with the current practice may not have been fully observable. However, the median duration of available medical history prior to GLP‐1RA initiation was substantial (19.5 years [IQR 9.5–30.6]), and the continuity of diabetes care within UK primary care makes it unlikely incomplete pre‐registration history meaningfully distorted observed treatment trajectories. Third, while we examined treatment lines, drug‐related side effects and reasons for treatment changes or discontinuation are not routinely captured in UK primary care records, limiting our ability to assess the drivers of observed treatment patterns. Fourth, our analyses were conducted at the level of glucose‐lowering drug classes rather than individual agents, which may obscure agent‐specific prescribing patterns. Fifth, some inconsistencies in prescribing patterns were observed that may reflect data recording inaccuracies or deviations from recommended practice. For instance, a subset of patients received DPP‐4is after initiating and while continuing GLP‐1RA therapy although both drug classes target the same incretin pathway and their concomitant use is not supported.^5^, ^6^ This overlap is likely attributable to transitional prescribing during therapy switching or to artefacts arising from overlapping prescription supply estimates rather than true concurrent use. Sixth, BMI‐stratified analyses relied on the availability of recent BMI measurements and may therefore be subject to selection bias. Finally, this study did not assess the clinical implications associated with different treatment trajectories, including short‐term outcomes such as glycaemic control and long‐term diabetes‐related complications, which may help explain observed prescribing patterns and warrants further investigation.

Overall, this population‐based study provides important real‐world insights into the trajectories and transitions between glucose‐lowering therapies surrounding GLP‐1RA initiation in UK primary care. While most individuals initiated GLP‐1RA therapy at later stages of type 2 diabetes management, typically after exposure to three or more different glucose‐lowering agents, consistent with contemporary UK clinical guidance (NG28),^8^ our findings reveal critical deviations that raise concerns about missed opportunities for early intervention in high‐risk populations. Specifically, individuals initiating GLP‐1RA therapy with a history of CVD were not more likely to receive SGLT‐2is, despite strong guideline support for their use in this population.^8^ Similarly, although semaglutide is endorsed by the NICE guidance on weight management irrespective of diabetes status due to its high weight‐loss efficacy,^19^ it was not preferentially prescribed among those with a BMI ≥35 kg/m^2^.

Cumulatively, these findings underscore the need for more effective implementation of clinical guidelines and reveal persistent treatment gaps in the UK, where access to GLP‐1RAs is determined by NICE guidelines within a publicly funded healthcare system, potentially delaying GLP‐1RA initiation among many high‐risk individuals compared with the more proactive approach endorsed by the ADA and EASD.^5^, ^6^, ^8^ Additionally, the strong alignment of GLP‐1RA prescribing with NICE guidance poses challenges for designing comparative effectiveness and safety studies, particularly those employing new‐user designs, due to the lack of suitable active comparators.

Nonetheless, research is needed to explore the real‐world impact of these treatment trajectories on clinical outcomes and quality of life in individuals with type 2 diabetes managed in the UK. Ongoing monitoring of evolving prescribing patterns and adherence to guidelines will be essential, not only to identify opportunities to optimise treatment strategies, but also to inform the design of clinical‐context sensitive real‐world comparative effectiveness and safety studies.

## AUTHOR CONTRIBUTIONS

AMB and FSU were responsible for study conception. AMB and FSU designed the study. FSU performed the statistical analyses. AMB, FSU, MFN, and NN contributed to the interpretation of the data. AMB and FSU wrote the manuscript. AMB, FSU, MFN, and NN contributed to important and critical edits of the manuscript draft and approved the final manuscript. AMB and FSU had full access to all the data in the study and take responsibility for the integrity of the data and the accuracy of the data analysis. The corresponding author attests that all listed authors meet authorship criteria and that no others meeting the criteria have been omitted.

## FUNDING INFORMATION

This study was funded by a Novartis Foundation for Medical‐Biological Research grant (grant number: 24B110). The funder had no role in study design, data collection, data analysis, data interpretation, writing of the report or in the decision to submit the article for publication.

## CONFLICT OF INTEREST STATEMENT

All authors declare no conflicts of interest and acknowledge support from the funder for the submitted work.

## ETHICS STATEMENT

The present study was approved by the Scientific Review Committee (23SRC017) from IQVIA, while ethical approval for using the IQVIA Medical Research Data (IMRD) incorporating data from THIN, a Cegedim Database, was granted by the NHS Health Research Authority (23/EM/0151).^11^

## Supporting information


**Data S1.** Supporting Information.

## Data Availability

Access to the database, the IQVIA Medical Research Data (IMRD) incorporating data from THIN, a Cegedim Database, used in this study was obtained through a purchase from IQVIA, and it cannot be redistributed or shared publicly without undergoing a review and obtaining consent from IQVIA. This precaution is needed due to the sensitive nature of the patient data.
