# Supplementary material for: Real‐world treatment trajectories preceding GLP‐1 receptor agonist initiation in type 2 diabetes: A descriptive UK population‐based cohort study on adherence to national clinical guidelines
Source: Diabetes Obes Metab. 2026 Feb 25;28(5):3692–702. doi: 10.1111/dom.70548 (PMC13071226; doi:10.1111/dom.70548)
Supplement: Supplementary file 1 — Data S1. Supporting Information. [file DOM-28-3692-s001.docx]

**Online-Only Supplementary Material**

Table of Contents

[SUPPLEMENTARY TABLES 3](#_Toc208085741)

[Table S1. ATC codes and generic names to define glucagon-like peptide-1 (GLP-1) receptor agonist use. 3](#_Toc208085742)

[Table S2. Classes of glucose-lowering therapies defined by ATC codes and agents. 4](#_Toc208085743)

[Table S3. Characteristics of individuals initiating glucagon-like peptide-1 (GLP-1) receptor agonist therapy between 01 January 2007 and 30 June 2023, stratified by the line of glucose-lowering therapy at GLP-1 receptor agonist initiation. 5](#_Toc208085744)

[Table S4. Characteristics of individuals initiating glucagon-like peptide-1 (GLP-1) receptor agonist therapy between 01 January 2007 and 31 December 2017, stratified by the line of glucose-lowering therapy at GLP-1 receptor agonist initiation. 7](#_Toc208085745)

[Table S5. Characteristics of individuals initiating glucagon-like peptide-1 (GLP-1) receptor agonist therapy between 01 January 2018 and 30 June 2023, stratified by the line of glucose-lowering therapy at GLP-1 receptor agonist initiation. 8](#_Toc208085746)

[Table S6. Glucose-lowering therapies from the first prescription up to glucagon-like peptide-1 (GLP-1) receptor agonist initiation between 01 January 2007 and 30 June 2023, stratified by treatment line*. 9](#_Toc208085747)

[Table S7. Glucose-lowering therapies (GLT)* used during the one year before and after glucagon-like peptide-1 (GLP-1) receptor agonist initiation, overall and stratified by initiation before 2018 and in 2018 or later. 10](#_Toc208085748)

[Table S8. Characteristics of individuals initiating glucagon-like peptide-1 (GLP-1) receptor agonist therapy between 01 January 2018 and 30 June 2023, stratified by cardiovascular disease (CVD) history. 11](#_Toc208085749)

[Table S9. Glucose-lowering therapies (GLT)* used during the one year before and after glucagon-like peptide-1 (GLP-1) receptor agonist initiation in 2018 or later, by subgroups of cardiovascular disease (CVD) history at initiation. 12](#_Toc208085750)

[Table S10. Glucose-lowering therapies from the first prescription up to glucagon-like peptide-1 (GLP-1) receptor agonist initiation between 01 January 2018 and 30 June 2023, stratified by treatment line* and cardiovascular disease (CVD) history at GLP-1 receptor agonist initiation. 13](#_Toc208085751)

[Table S11. Characteristics of individuals initiating glucagon-like peptide-1 (GLP-1) receptor agonist therapy between 01 January 2018 and 30 June 2023, stratified by body-mass index (BMI)§ at initiation. 14](#_Toc208085752)

[Table S12. Glucose-lowering therapies (GLT)* used during the one year before and after glucagon-like peptide-1 (GLP-1) receptor agonist initiation in 2018 or later, stratified by body-mass index (BMI)† category at initiation. 15](#_Toc208085753)

[Table S13. Glucose-lowering therapies from the first prescription up to glucagon-like peptide-1 (GLP-1) receptor agonist initiation between 01 January 2018 and 30 June 2023, stratified by treatment line* and by body-mass index (BMI)† category at initiation. 16](#_Toc208085754)

[Table S14. Characteristics of individuals initiating glucagon-like peptide-1 (GLP-1) receptor agonist therapy between 01 January 2018 and 30 June 2023, stratified by sex assigned at birth. 17](#_Toc208085755)

[Table S15. Glucose-lowering therapies (GLT)* used during the one year before and after glucagon-like peptide-1 (GLP-1) receptor agonist initiation in 2018 or later, stratified by sex assigned at birth. 18](#_Toc208085756)

[Table S16. Glucose-lowering therapies from the first prescription up to glucagon-like peptide-1 (GLP-1) receptor agonist initiation between 01 January 2018 and 30 June 2023, stratified by treatment line* and sex assigned at birth. 19](#_Toc208085757)

[SUPPLEMENTARY FIGURES 20](#_Toc208085758)

[Figure S1. Cohort construction of individuals initiating glucagon-like peptide-1 (GLP-1) receptor agonist therapy using the IQVIA Medical Research Data (IMRD) incorporating data from THIN, A Cegedim Database. 20](#_Toc208085759)

[Figure S2. Sankey diagram showing the trajectories of glucose-lowering therapies from the first prescription to the initiation of glucagon-like peptide-1 (GLP-1) receptor agonist therapy among individuals initiating GLP-1 receptor agonist therapy between 01-Jan-2007 to 30-Jun-2023, stratified by treatment line*. 21](#_Toc208085760)

[Figure S3. Sankey diagram showing the trajectories of glucose-lowering therapies from the first prescription to the initiation of glucagon-like peptide-1 (GLP-1) receptor agonist therapy among individuals initiating GLP-1 receptor agonist therapy between 01-Jan-2018 to 30-Jun-2023, stratified by treatment line* and cardiovascular disease (CVD) history at GLP-1 receptor agonist initiation (A, B). 22](#_Toc208085761)

[Figure S4. Sankey diagram showing the trajectories of glucose-lowering therapies from the first prescription to the initiation of glucagon-like peptide-1 (GLP-1) receptor agonist therapy among individuals initiating GLP-1 receptor agonist therapy between 01-Jan-2018 to 30-Jun-2023, stratified by treatment line* and body-mass index (BMI)† at GLP-1 receptor agonist initiation (A, B). 23](#_Toc208085762)

[Figure S5. Sankey diagram showing the trajectories of glucose-lowering therapies from the first prescription to the initiation of glucagon-like peptide-1 (GLP-1) receptor agonist therapy among individuals initiating GLP-1 receptor agonist therapy between 01-Jan-2018 to 30-Jun-2023, stratified by treatment line* and sex assigned at birth (A, B). 24](#_Toc208085763)

SUPPLEMENTARY TABLES

| **Table S1. ATC codes and generic names to define glucagon-like peptide-1 (GLP-1) receptor agonist use.** | | |
| --- | --- | --- |
| **GLP-1 receptor agonist (ATC code)** | **Generic name** | **Combination product with insulin** |
| **Semaglutide (A10BJ06)** | Rybelsus 3mg tablets | No |
|  | Semaglutide 3mg tablets | No |
|  | Rybelsus 7mg tablets | No |
|  | Semaglutide 7mg tablets | No |
|  | Rybelsus 14mg tablets | No |
|  | Semaglutide 14mg tablets | No |
|  | Ozempic 1mg/0.74ml solution for injection 3ml pre-filled pen | No |
|  | Semaglutide 1mg/0.74ml solution for injection 3ml pre-filled disposable device | No |
|  | Ozempic 0.5mg/0.37ml solution for injection 1.5ml pre-filled pen | No |
|  | Semaglutide 0.5mg/0.37ml solution for injection 1.5ml pre-filled disposable device | No |
|  | Ozempic 0.25mg/0.19ml solution for injection 1.5ml pre-filled pen | No |
|  | Semaglutide 0.25mg/0.19ml solution for injection 1.5ml pre-filled disposable device | No |
| **Dulaglutide (A10BJ05)** | Trulicity 3mg/0.5ml solution for injection pre-filled pens | No |
|  | Dulaglutide 3mg/0.5ml solution for injection pre-filled disposable devices | No |
|  | Trulicity 4.5mg/0.5ml solution for injection pre-filled pens | No |
|  | Dulaglutide 4.5mg/0.5ml solution for injection pre-filled disposable devices | No |
|  | Dulaglutide 1.5mg/0.5ml solution for injection pre-filled disposable devices | No |
|  | Dulaglutide 0.75mg/0.5ml solution for injection pre-filled disposable devices | No |
| **Liraglutide (A10BJ02)** | Liraglutide 6mg/ml solution for injection 3ml pre-filled disposable devices | No |
|  | Insulin degludec 100units/ml / Liraglutide 3.6mg/ml solution for injection 3ml pre-filled disposable devices | Yes |
| **Exenatide (A10BJ01)** | Bydureon BCise 2mg/0.85ml prolonged-release suspension for injection pre-filled pens | No |
|  | Exenatide 2mg/0.85ml prolonged-release suspension for injection pre-filled disposable devices | No |
|  | Exenatide 2mg powder and solvent for prolonged-release suspension for injection pre-filled disposable devices | No |
|  | Exenatide 2mg injection | No |
|  | Exenatide 2mg powder and solvent for prolonged-release suspension for injection vials | No |
|  | Exenatide 10micrograms/0.04ml solution for injection 2.4ml pre-filled disposable devices | No |
|  | Exenatide 5micrograms/0.02ml solution for injection 1.2ml pre-filled disposable devices | No |
| **Lixisenatide (A10BJ03)** | Lixisenatide 10micrograms/0.2ml solution for injection 3ml pre-filled disposable devices | No |
|  | Lixisenatide 10micrograms/0.2ml solution for injection 3ml pre-filled disposable devices and Lixisenatide 20micrograms/0 | No |
|  | Lixisenatide 20micrograms/0.2ml solution for injection 3ml pre-filled disposable devices | No |
|  | Insulin glargine 100units/ml / Lixisenatide 33micrograms/ml solution for injection 3ml pre-filled disposable devices | Yes |
|  | Suliqua 100units/ml / 50micrograms/ml solution for injection 3ml pre-filled SoloStar pen | Yes |
|  | Suliqua 100units/ml / 33micrograms/ml solution for injection 3ml pre-filled SoloStar pen | Yes |
| **Albiglutide (A10BJ04)** | Albiglutide 30mg powder and solvent for solution for injection pre-filled disposable devices | No |

| **Table S2. Classes of glucose-lowering therapies defined by ATC codes and agents.** | | | |
| --- | --- | --- | --- |
| **Drug class** | **Drug class ATC code** | **Agent** | **Agent ATC code** |
| Biguanides | A10BA | Metformin | A10BA02 |
| Sulfonylureas | A10BB | Glibenclamide | A10BB01 |
|  |  | Chlorpropamide | A10BB02 |
|  |  | Tolbutamide | A10BB03 |
|  |  | Tolazamide | A10BB05 |
|  |  | Gliclazide | A10BB09 |
|  |  | Glimepiride | A10BB12 |
| Thiazolidinediones | A10BG | Troglitazone | A10BG01 |
|  |  | Rosiglitazone | A10BG02 |
|  |  | Pioglitazone | A10BG03 |
| Dipeptidyl peptidase 4 (DPP-4) inhibitors | A10BH | Sitagliptin | A10BH01 |
|  |  | Vildagliptin | A10BH02 |
|  |  | Saxagliptin | A10BH03 |
|  |  | Alogliptin | A10BH04 |
|  |  | Linagliptin | A10BH05 |
| Glucagon-like peptide-1 (GLP-1) receptor agonists | A10BJ | Exenatide | A10BJ01 |
|  |  | Liraglutide | A10BJ02 |
|  |  | Lixisenatide | A10BJ03 |
|  |  | Albiglutide | A10BJ04 |
|  |  | Dulaglutide | A10BJ05 |
|  |  | Semaglutide | A10BJ06 |
| Sodium-glucose co-transporter 2 (SGLT-2) inhibitors | A10BK | Dapagliflozin | A10BK01 |
|  |  | Canagliflozin | A10BK02 |
|  |  | Empagliflozin | A10BK03 |
|  |  | Ertugliflozin | A10BK04 |
| Insulin | A10A | All agents identified by an ATC code starting with “A10A” | ATC codes starting with “A10A” |
| Other glucose-lowering drugs | Not applicable | Acarbose | A10BF01 |
|  |  | Repaglinide | A10BX02 |
|  |  | Nateglinide | A10BX03 |
|  |  | Guar gum | A10BX01 |

| **Table S3. Characteristics of individuals initiating glucagon-like peptide-1 (GLP-1) receptor agonist therapy between 01 January 2007 and 30 June 2023, stratified by the line of glucose-lowering therapy at GLP-1 receptor agonist initiation.** | | | | | | | |
| --- | --- | --- | --- | --- | --- | --- | --- |
| **Characteristics** | **Overall** | **GLP-1 receptor agonist as…** | | | | | |
|  |  | **First-line** | **Second-line** | **Third-line** | **Fourth-line** | **Fifth-line** | **Sixth to eighth-line** |
| Individuals included, n (%) | 29780 (100.0) | 221 (0.8) | 3126 (10.5) | 7508 (25.2) | 10547 (35.4) | 6492 (21.8) | 1886 (6.3) |
| Age at initiation, years, median (IQR) | 59.0 [51.5, 66.3] | 54.9 [46.9, 60.8] | 54.9 [47.4, 62.7] | 56.9 [49.6, 64.5] | 59.2 [51.9, 66.4] | 61.6 [54.3, 68.3] | 63.2 [56.0, 70.2] |
| Female sex assigned at birth, n (%) | 13157 (44.2) | 121 (54.8) | 1469 (47.0) | 3311 (44.1) | 4599 (43.6) | 2827 (43.5) | 830 (44.0) |
| Type 2 diabetes duration, years, median (IQR) | 6.4 [3.4, 10.3] | 0.00 [0.0, 0.0] | 2.10 [0.9, 4.0] | 4.30 [2  .4, 7.0] | 6.80 [4.3, 9.9] | 9.6 [6.6, 12.8] | 13.0 [9.5, 16.2] |
| **GLP-1 receptor agonist agent initiated, n (%)** | | | | | | | |
| Semaglutide | 5084 (17.1) | 92 (41.6) | 718 (23.0) | 1306 (17.4) | 1506 (14.3) | 1066 (16.4) | 396 (21.0) |
| Dulaglutide | 5665 (19.0) | 44 (19.9) | 639 (20.4) | 1318 (17.6) | 1823 (17.3) | 1368 (21.1) | 473 (25.1) |
| Liraglutide | 10017 (33.6) | 57 (25.8) | 956 (30.6) | 2417 (32.2) | 3700 (35.1) | 2264 (34.9) | 623 (33.0) |
| Exenatide | 7912 (26.6) | 26 (11.8) | 705 (22.6) | 2212 (29.5) | 3085 (29.3) | 1561 (24.0) | 323 (17.1) |
| Lixisenatide | 1102 (3.7) | <7 | 108 (3.5) | 255 (3.4) | 433 (4.1) | 233 (3.6) | 71 (3.8) |
| **Previous use of glucose-lowering therapies*, n (%)** | | | | | | | |
| Metformin | 29289 (98.4) | <7 | 3030 (96.9) | 7397 (98.5) | 10492 (99.5) | 6484 (99.9) | 1886 (100.0) |
| Sulfonylureas | 20267 (68.1) | <7 | 520 (16.6) | 3648 (48.6) | 8109 (76.9) | 6126 (94.4) | 1864 (98.8) |
| SGLT-2 inhibitors | 8093 (27.2) | <7 | 169 (5.4) | 1624 (21.6) | 2679 (25.4) | 2538 (39.1) | 1083 (57.4) |
| DPP-4 inhibitors | 14823 (49.8) | <7 | 205 (6.6) | 2198 (29.3) | 5919 (56.1) | 4879 (75.2) | 1622 (86.0) |
| Thiazolidinediones | 10060 (33.8) | <7 | 143 (4.6) | 1116 (14.9) | 3524 (33.4) | 3678 (56.7) | 1599 (84.8) |
| Insulin | 5345 (17.9) | <7 | <7 | 592 (7.9) | 1634 (15.5) | 2024 (31.2) | 1095 (58.1) |
| Other glucose-lowering drugs | 1386 (4.7) | <7 | <7 | 60 (0.8) | 242 (2.3) | 524 (8.1) | 554 (29.4) |
| **Concurrent use of glucose-lowering therapies†, n (%)** | | | | | | | |
| Metformin | 26197 (88.0) | 67 (30.3) | 2930 (93.7) | 6846 (91.2) | 9278 (88.0) | 5520 (85.0) | 1556 (82.5) |
| Sulfonylureas | 15053 (50.5) | 10 (4.5) | 507 (16.2) | 3231 (43.0) | 6381 (60.5) | 4000 (61.6) | 924 (49.0) |
| SGLT-2 inhibitors | 6828 (22.9) | 13 (5.9) | 179 (5.7) | 1523 (20.3) | 2317 (22.0) | 2011 (31.0) | 785 (41.6) |
| DPP-4 inhibitors | 12019 (40.4) | <7 | 198 (6.3) | 2031 (27.1) | 5169 (49.0) | 3655 (56.3) | 965 (51.2) |
| Thiazolidinediones | 4411 (14.8) | <7 | 120 (3.8) | 837 (11.1) | 2122 (20.1) | 1057 (16.3) | 275 (14.6) |
| Insulin | 4878 (16.4) | <7 | 12 (0.4) | 569 (7.6) | 1525 (14.5) | 1842 (28.4) | 930 (49.3) |
| Other glucose-lowering drugs | 354 (1.2) | <7 | <7 | 37 (0.5) | 110 (1.0) | 121 (1.9) | 81 (4.3) |
| **Comorbidities‡, n (%)** | | | | | | | |
| Cardiovascular disease | 5459 (18.3) | 35 (15.8) | 459 (14.7) | 1307 (17.4) | 1934 (18.3) | 1298 (20.0) | 426 (22.6) |
| Hypertension | 19699 (66.1) | 124 (56.1) | 1863 (59.6) | 4700 (62.6) | 7089 (67.2) | 4591 (70.7) | 1332 (70.6) |
| Dyslipidemia | 6583 (22.1) | 21 (9.5) | 522 (16.7) | 1477 (19.7) | 2416 (22.9) | 1668 (25.7) | 479 (25.4) |
| Depression | 11310 (38.0) | 109 (49.3) | 1286 (41.1) | 2919 (38.9) | 3917 (37.1) | 2372 (36.5) | 707 (37.5) |
| Asthma | 6536 (21.9) | 62 (28.1) | 747 (23.9) | 1718 (22.9) | 2244 (21.3) | 1359 (20.9) | 406 (21.5) |
| Chronic obstructive pulmonary disease | 7522 (25.3) | 66 (29.9) | 811 (25.9) | 1929 (25.7) | 2660 (25.2) | 1600 (24.6) | 456 (24.2) |
| Chronic kidney disease | 4569 (15.3) | 26 (11.8) | 320 (10.2) | 951 (12.7) | 1647 (15.6) | 1207 (18.6) | 418 (22.2) |
| Osteoarthritis | 6558 (22.0) | 41 (18.6) | 620 (19.8) | 1508 (20.1) | 2323 (22.0) | 1565 (24.1) | 501 (26.6) |
| **Laboratory and vital sign measurements§** | | | | | | | |
| BMI, kg/m^2^, median (IQR) | 35.4 [31.8, 39.3] | 39.3 [35.0, 43.0] | 37.7 [33.9, 41.7] | 36.1 [32.7, 40.1] | 35.3 [31.9, 39.0] | 34.3 [30.9, 38.2] | 33.1 [30.1, 37.1] |
| BMI ≥35, n (%) | 12419 (41.7) | 84 (38.0) | 1463 (46.8) | 3435 (45.8) | 4461 (42.3) | 2391 (36.8) | 585 (31.0) |
| BMI missing, n (%) | 6286 (21.1) | 109 (49.3) | 946 (30.3) | 1703 (22.7) | 1998 (18.9) | 1180 (18.2) | 350 (18.6) |
| eGFR, ml/min per 1.72m^2^, median (IQR) | 98.9 [83.8, 108.2] | 102.85 [86.45, 112.22] | 103.20 [91.20, 112.30] | 101.00 [87.60, 109.80] | 98.50 [82.97, 107.60] | 96.30 [79.30, 105.60] | 94.30 [75.90, 103.60] |
| eGFR <60, n (%) | 2201 (7.4) | 16 (7.2) | 127 (4.1) | 469 (6.2) | 787 (7.5) | 599 (9.2) | 203 (10.8) |
| Missing eGFR, n (%) | 900 (3.0) | 31 (14.0) | 111 (3.6) | 217 (2.9) | 295 (2.8) | 188 (2.9) | 58 (3.1) |
| HbA1c, %, median (IQR) | 8.19 [7.73, 8.55] | 6.91 [6.54, 7.37] | 7.82 [7.18, 8.37] | 8.19 [7.64, 8.55] | 8.28 [7.82, 8.65] | 8.28 [7.82, 8.65] | 8.28 [7.82, 8.65] |
| Abbreviations: GLP-1 glucagon-like peptide-1, SGLT-2 sodium-glucose co-transporter 2, DPP-4 dipeptidyl peptidase-4, HbA1c glycated hemoglobin, BMI body-mass index, eGFR estimated glomerular filtration rate. *Defined as having at least one prescription for a glucose-lowering therapy specified in Supplementary Table S2 before GLP-1 receptor agonist initiation. †Defined as having at least one prescription for a glucose-lowering therapy specified in Supplementary Table S2 within the 180 days before or at GLP-1 receptor agonist initiation. ‡Diagnosis recorded before or at GLP-1 receptor agonist initiation. §Most recent measurement recorded within the year before or at GLP-1 receptor agonist initiation. | | | | | | | |

| **Table S4. Characteristics of individuals initiating glucagon-like peptide-1 (GLP-1) receptor agonist therapy between 01 January 2007 and 31 December 2017, stratified by the line of glucose-lowering therapy at GLP-1 receptor agonist initiation.** | | | | | | | | | | | |
| --- | --- | --- | --- | --- | --- | --- | --- | --- | --- | --- | --- |
| **Characteristics** | **Initiation pre-2018** | **GLP-1 receptor agonist as…** | | | | | | | | | |
|  |  | **First-line** | **Second-line** | | | **Third-line** | **Fourth-line** | | **Fifth-line** | | **Sixth to eighth-line** |
| Individuals included, n (%) | 18517 (100.0) | 61 (0.3) | | 1663 (9.0) | 4795 (25.9) | | 7072 (38.2) | 3973 (21.5) | | 953 (5.1) | |
| Age at initiation, years, median (IQR) | 58.0 [50.6, 65.0] | 54.4 [46.0, 61.7] | | 53.70 [46.5, 61.6] | 55.8 [48.9, 63.4] | | 58.3 [51.3, 65.2] | 60.5 [53.5, 67.0] | | 61.3 [54.0, 68.0] | |
| Female sex assigned at birth, n (%) | 8123 (43.9) | 26 (42.6) | | 753 (45.3) | 2118 (44.2) | | 3039 (43.0) | 1753 (44.1) | | 434 (45.5) | |
| Type 2 diabetes duration, years, median (IQR) | 6.3 [3.5, 9.7] | 0.0 [0.0, 0.0] | | 2.2 [1.1, 4.0] | 4.4 [2.5, 7.0] | | 6.7 [4.3, 9.6] | 9.1 [6.4, 12.0] | | 11.4 [8.5, 14.2] | |
| **GLP-1 receptor agonist agent initiated, n (%)** | | | | | | | | | | | |
| Semaglutide | <7 | <7 | | <7 | <7 | | <7 | <7 | | <7 | |
| Dulaglutide | 1003 (5.4) | <7 | | 73 (4.4) | 218 (4.5) | | 340 (4.8) | 271 (6.8) | | 98 (10.3) | |
| Liraglutide | 8783 (47.4) | 30 (49.2) | | 821 (49.4) | 2174 (45.3) | | 3316 (46.9) | 1954 (49.2) | | 488 (51.2) | |
| Exenatide | 7671 (41.4) | 26 (42.6) | | 663 (39.9) | 2156 (45.0) | | 3009 (42.5) | 1520 (38.3) | | 297 (31.2) | |
| Lixisenatide | 1060 (5.7) | <7 | | 106 (6.4) | 247 (5.2) | | 407 (5.8) | 228 (5.7) | | 70 (7.3) | |
| **Previous use of glucose-lowering therapies*, n (%)** | | | | | | | | | | | |
| Metformin | 18357 (99.1) | <7 | | 1638 (98.5) | 4747 (99.0) | | 7048 (99.7) | 3971 (99.9) | | 953 (100.0) | |
| Sulfonylureas | 13981 (75.5) | <7 | | 333 (20.0) | 2815 (58.7) | | 6079 (86.0) | 3804 (95.7) | | 950 (99.7) | |
| SGLT-2 inhibitors | 1266 (6.8) | <7 | | 7 (0.4) | 120 (2.5) | | 373 (5.3) | 498 (12.5) | | 268 (28.1) | |
| DPP-4 inhibitors | 8367 (45.2) | <7 | | 74 (4.4) | 1367 (28.5) | | 3542 (50.1) | 2645 (66.6) | | 739 (77.5) | |
| Thiazolidinediones | 8335 (45.0) | <7 | | 130 (7.8) | 1038 (21.6) | | 3198 (45.2) | 3094 (77.9) | | 875 (91.8) | |
| Insulin | 3897 (21.0) | <7 | | <7 | 409 (8.5) | | 1310 (18.5) | 1551 (39.0) | | 627 (65.8) | |
| Other glucose-lowering drugs | 1254 (6.8) | <7 | | <7 | 56 (1.2) | | 229 (3.2) | 494 (12.4) | | 470 (49.3) | |
| **Concurrent use of glucose-lowering therapies†, n (%)** | | | | | | | | | | | |
| Metformin | 16662 (90.0) | 16 (26.2) | | 1595 (95.9) | 4475 (93.3) | | 6355 (89.9) | 3424 (86.2) | | 797 (83.6) | |
| Sulfonylureas | 10704 (57.8) | <7 | | 328 (19.7) | 2554 (53.3) | | 4853 (68.6) | 2504 (63.0) | | 460 (48.3) | |
| SGLT-2 inhibitors | 1105 (6.0) | <7 | | 11 (0.7) | 121 (2.5) | | 326 (4.6) | 429 (10.8) | | 217 (22.8) | |
| DPP-4 inhibitors | 7117 (38.4) | <7 | | 71 (4.3) | 1295 (27.0) | | 3184 (45.0) | 2089 (52.6) | | 478 (50.2) | |
| Thiazolidinediones | 3911 (21.1) | <7 | | 109 (6.6) | 793 (16.5) | | 1965 (27.8) | 884 (22.3) | | 160 (16.8) | |
| Insulin | 3574 (19.3) | <7 | | <7 | 392 (8.2) | | 1225 (17.3) | 1415 (35.6) | | 536 (56.2) | |
| Other glucose-lowering drugs | 327 (1.8) | <7 | | <7 | 34 (0.7) | | 105 (1.5) | 115 (2.9) | | 68 (7.1) | |
| **Comorbidities‡, n (%)** | | | | | | | | | | | |
| Cardiovascular disease | 3356 (18.1) | 12 (19.7) | | 253 (15.2) | 810 (16.9) | | 1280 (18.1) | 794 (20.0) | | 207 (21.7) | |
| Hypertension | 12525 (67.6) | 38 (62.3) | | 1043 (62.7) | 3044 (63.5) | | 4849 (68.6) | 2860 (72.0) | | 691 (72.5) | |
| Dyslipidemia | 4394 (23.7) | <7 | | 302 (18.2) | 996 (20.8) | | 1732 (24.5) | 1086 (27.3) | | 274 (28.8) | |
| Depression | 6690 (36.1) | 24 (39.3) | | 652 (39.2) | 1754 (36.6) | | 2521 (35.6) | 1382 (34.8) | | 357 (37.5) | |
| Asthma | 3996 (21.6) | 18 (29.5) | | 403 (24.2) | 1076 (22.4) | | 1452 (20.5) | 837 (21.1) | | 210 (22.0) | |
| Chronic obstructive pulmonary disease | 4631 (25.0) | 19 (31.1) | | 427 (25.7) | 1214 (25.3) | | 1744 (24.7) | 991 (24.9) | | 236 (24.8) | |
| Chronic kidney disease | 2795 (15.1) | 12 (19.7) | | 183 (11.0) | 599 (12.5) | | 1051 (14.9) | 752 (18.9) | | 198 (20.8) | |
| Osteoarthritis | 3901 (21.1) | 9 (14.8) | | 317 (19.1) | 927 (19.3) | | 1496 (21.2) | 905 (22.8) | | 247 (25.9) | |
| **Laboratory and vital sign measurements§** | | | | | | | | | | | |
| BMI, kg/m^2^, median (IQR) | 36.10 [32.70, 40.00] | 38.55 [34.12, 40.00] | | 38.00 [34.50, 41.80] | 36.80 [33.50, 40.60] | | 36.00 [32.65, 39.70] | 35.40 [32.10, 39.10] | | 34.10 [31.30, 38.30] | |
| BMI ≥35, n (%) | 8765 (47.3) | 23 (37.7) | | 835 (50.2) | 2435 (50.8) | | 3360 (47.5) | 1747 (44.0) | | 365 (38.3) | |
| BMI missing, n (%) | 3590 (19.4) | 29 (47.5) | | 482 (29.0) | 1027 (21.4) | | 1225 (17.3) | 674 (17.0) | | 153 (16.1) | |
| eGFR, ml/min per 1.72m^2^, median (IQR) | 99.40 [84.70, 108.40] | 100.65 [79.30, 112.45] | | 103.50 [91.80, 112.60] | 101.60 [88.60, 110.00] | | 98.90 [84.10, 107.80] | 96.60 [79.73, 105.80] | | 95.00 [78.90, 105.20] | |
| eGFR <60, n (%) | 1126 (6.1) | <7 | | 58 (3.5) | 233 (4.9) | | 416 (5.9) | 331 (8.3) | | 82 (8.6) | |
| Missing eGFR, n (%) | 566 (3.1) | 11 (18.0) | | 68 (4.1) | 146 (3.0) | | 194 (2.7) | 119 (3.0) | | 28 (2.9) | |
| HbA1c, %, median (IQR) | 8.19 [7.73, 8.55] | 6.72 [6.18, 7.32] | | 7.91 [7.27, 8.46] | 8.19 [7.73, 8.55] | | 8.28 [7.82, 8.65] | 8.28 [7.82, 8.55] | | 8.28 [7.73, 8.65] | |
| Abbreviations: GLP-1 glucagon-like peptide-1, SGLT-2 sodium-glucose co-transporter 2, DPP-4 dipeptidyl peptidase-4, HbA1c glycated hemoglobin, BMI body-mass index, eGFR estimated glomerular filtration rate. *Defined as having at least one prescription for a glucose-lowering therapy specified in Supplementary Table S2 before GLP-1 receptor agonist initiation. †Defined as having at least one prescription for a glucose-lowering therapy specified in Supplementary Table S2 within the 180 days before or at GLP-1 receptor agonist initiation. ‡Diagnosis recorded before or at GLP-1 receptor agonist initiation. §Most recent measurement recorded within the year before or at GLP-1 receptor agonist initiation. | | | | | | | | | | | |

| **Table S5. Characteristics of individuals initiating glucagon-like peptide-1 (GLP-1) receptor agonist therapy between 01 January 2018 and 30 June 2023, stratified by the line of glucose-lowering therapy at GLP-1 receptor agonist initiation.** | | | | | | | |
| --- | --- | --- | --- | --- | --- | --- | --- |
| **Characteristics** | **Initiation post-2018** | **GLP-1 receptor agonist as…** | | | | | |
|  |  | **First-line** | **Second-line** | **Third-line** | **Fourth-line** | **Fifth-line** | **Sixth to eighth-line** |
| Individuals included, n (%) | 11263 (100.0) | 160 (1.4) | 1463 (13.0) | 2713 (24.1) | 3475 (30.8) | 2519 (22.4) | 933 (8.3) |
| Age at initiation, years, median (IQR) | 60.8 [53.1, 68.6] | 55.2 [47.5, 60.6] | 56.1 [48.8, 64.2] | 58.6 [51.4, 66.8] | 61.1 [53.7, 68.9] | 63.2 [56.1, 70.4] | 65.4 [58.7, 71.9] |
| Female sex assigned at birth, n (%) | 5034 (44.7) | 95 (59.4) | 716 (48.9) | 1193 (44.0) | 1560 (44.9) | 1074 (42.6) | 396 (42.4) |
| Type 2 diabetes duration, years, median (IQR) | 6.7 [3.2, 11.5] | 0.0 [0.0, 0.0] | 2.0 [0.8, 4.1] | 4.2 [2.2, 7.0] | 7.1 [4.3, 10.9] | 10.5 [7.1, 14.1] | 14.9 [11.1, 18.3] |
| **GLP-1 receptor agonist agent initiated, n (%)** | | | | | | | |
| Semaglutide | 5084 (45.1) | 92 (57.5) | 718 (49.1) | 1306 (48.1) | 1506 (43.3) | 1066 (42.3) | 396 (42.4) |
| Dulaglutide | 4662 (41.4) | 41 (25.6) | 566 (38.7) | 1100 (40.5) | 1483 (42.7) | 1097 (43.5) | 375 (40.2) |
| Liraglutide | 1234 (11.0) | 27 (16.9) | 135 (9.2) | 243 (9.0) | 384 (11.1) | 310 (12.3) | 135 (14.5) |
| Exenatide | 241 (2.1) | <7 | 42 (2.9) | 56 (2.1) | 76 (2.2) | 41 (1.6) | 26 (2.8) |
| Lixisenatide | 42 (0.4) | <7 | <7 | 8 (0.3) | 26 (0.7) | <7 | <7 |
| **Previous use of glucose-lowering therapies*, n (%)** | | | | | | | |
| Metformin | 10932 (97.1) | <7 | 1392 (95.1) | 2650 (97.7) | 3444 (99.1) | 2513 (99.8) | 933 (100.0) |
| Sulfonylureas | 6286 (55.8) | <7 | 187 (12.8) | 833 (30.7) | 2030 (58.4) | 2322 (92.2) | 914 (98.0) |
| SGLT-2 inhibitors | 6827 (60.6) | <7 | 162 (11.1) | 1504 (55.4) | 2306 (66.4) | 2040 (81.0) | 815 (87.4) |
| DPP-4 inhibitors | 6456 (57.3) | <7 | 131 (9.0) | 831 (30.6) | 2377 (68.4) | 2234 (88.7) | 883 (94.6) |
| Thiazolidinediones | 1725 (15.3) | <7 | 13 (0.9) | 78 (2.9) | 326 (9.4) | 584 (23.2) | 724 (77.6) |
| Insulin | 1448 (12.9) | <7 | <7 | 183 (6.7) | 324 (9.3) | 473 (18.8) | 468 (50.2) |
| Other glucose-lowering drugs | 132 (1.2) | <7 | <7 | <7 | 13 (0.4) | 30 (1.2) | 84 (9.0) |
| **Concurrent use of glucose-lowering therapies†, n (%)** | | | | | | | |
| Metformin | 9535 (84.7) | 51 (31.9) | 1335 (91.3) | 2371 (87.4) | 2923 (84.1) | 2096 (83.2) | 759 (81.4) |
| Sulfonylureas | 4349 (38.6) | <7 | 179 (12.2) | 677 (25.0) | 1528 (44.0) | 1496 (59.4) | 464 (49.7) |
| SGLT-2 inhibitors | 5723 (50.8) | 12 (7.5) | 168 (11.5) | 1402 (51.7) | 1991 (57.3) | 1582 (62.8) | 568 (60.9) |
| DPP-4 inhibitors | 4902 (43.5) | <7 | 127 (8.7) | 736 (27.1) | 1985 (57.1) | 1566 (62.2) | 487 (52.2) |
| Thiazolidinediones | 500 (4.4) | <7 | 11 (0.8) | 44 (1.6) | 157 (4.5) | 173 (6.9) | 115 (12.3) |
| Insulin | 1304 (11.6) | <7 | <7 | 177 (6.5) | 300 (8.6) | 427 (17.0) | 394 (42.2) |
| Other glucose-lowering drugs | 27 (0.2) | <7 | <7 | <7 | <7 | <7 | 13 (1.4) |
| **Comorbidities‡, n (%)** | | | | | | | |
| Cardiovascular disease | 2103 (18.7) | 23 (14.4) | 206 (14.1) | 497 (18.3) | 654 (18.8) | 504 (20.0) | 219 (23.5) |
| Hypertension | 7174 (63.7) | 86 (53.8) | 820 (56.0) | 1656 (61.0) | 2240 (64.5) | 1731 (68.7) | 641 (68.7) |
| Dyslipidemia | 2189 (19.4) | 17 (10.6) | 220 (15.0) | 481 (17.7) | 684 (19.7) | 582 (23.1) | 205 (22.0) |
| Depression | 4620 (41.0) | 85 (53.1) | 634 (43.3) | 1165 (42.9) | 1396 (40.2) | 990 (39.3) | 350 (37.5) |
| Asthma | 2540 (22.6) | 44 (27.5) | 344 (23.5) | 642 (23.7) | 792 (22.8) | 522 (20.7) | 196 (21.0) |
| Chronic obstructive pulmonary disease | 2891 (25.7) | 47 (29.4) | 384 (26.2) | 715 (26.4) | 916 (26.4) | 609 (24.2) | 220 (23.6) |
| Chronic kidney disease | 1774 (15.8) | 14 (8.8) | 137 (9.4) | 352 (13.0) | 596 (17.2) | 455 (18.1) | 220 (23.6) |
| Osteoarthritis | 2657 (23.6) | 32 (20.0) | 303 (20.7) | 581 (21.4) | 827 (23.8) | 660 (26.2) | 254 (27.2) |
| **Laboratory and vital sign measurements§** | | | | | | | |
| BMI, kg/m^2^, median (IQR) | 34.0 [30.5, 38.0] | 39.7 [36.3, 44.9] | 37.3 [32.9, 41.5] | 34.9 [31.4, 38.8] | 33.8 [30.5, 37.5] | 32.5 [29.4, 36.3] | 31.8 [29.1, 35.6] |
| BMI ≥35, n (%) | 3654 (32.4) | 61 (38.1) | 628 (42.9) | 1000 (36.9) | 1101 (31.7) | 644 (25.6) | 220 (23.6) |
| BMI missing, n (%) | 2696 (23.9) | 80 (50.0) | 464 (31.7) | 676 (24.9) | 773 (22.2) | 506 (20.1) | 197 (21.1) |
| eGFR, ml/min per 1.72m^2^, median (IQR) | 98.2 [82.0, 107.7] | 103.2 [89.9, 112.0] | 102.8 [90.2, 111.7] | 99.7 [85.6, 109.4] | 97.9 [79.5, 107.2] | 95.9 [78.6, 105.0] | 93.1 [74.1, 102.3] |
| eGFR <60, n (%) | 1075 (9.5) | 10 (6.2) | 69 (4.7) | 236 (8.7) | 371 (10.7) | 268 (10.6) | 121 (13.0) |
| Missing eGFR, n (%) | 334 (3.0) | 20 (12.5) | 43 (2.9) | 71 (2.6) | 101 (2.9) | 69 (2.7) | 30 (3.2) |
| HbA1c, %, median (IQR) | 8.19 [7.64, 8.55] | 6.91 [6.63, 7.37] | 7.73 [7.00, 8.37] | 8.10 [7.55, 8.55] | 8.28 [7.73, 8.65] | 8.37 [7.91, 8.65] | 8.37 [7.82, 8.65] |
| Abbreviations: GLP-1 glucagon-like peptide-1, SGLT-2 sodium-glucose co-transporter 2, DPP-4 dipeptidyl peptidase-4, HbA1c glycated hemoglobin, BMI body-mass index, eGFR estimated glomerular filtration rate. *Defined as having at least one prescription for a glucose-lowering therapy specified in Supplementary Table S2 before GLP-1 receptor agonist initiation. †Defined as having at least one prescription for a glucose-lowering therapy specified in Supplementary Table S2 within the 180 days before or at GLP-1 receptor agonist initiation. ‡Diagnosis recorded before or at GLP-1 receptor agonist initiation. §Most recent measurement recorded within the year before or at GLP-1 receptor agonist initiation. | | | | | | | |

| **Table S6. Glucose-lowering therapies from the first prescription up to glucagon-like peptide-1 (GLP-1) receptor agonist initiation between 01 January 2007 and 30 June 2023, stratified by treatment line*.** | | | | | | | | |
| --- | --- | --- | --- | --- | --- | --- | --- | --- |
| **Glucose-lowering therapy** | **First-line** | **Second-line** | **Third-line** | **Fourth-line** | **Fifth-line** | **Sixth-line** | **Seventh-line** | **Eighth-line** |
| **Individuals initiating GLP-1 receptor agonist therapy between 01 January 2007 and 30 June 2023** | | | | | | | | |
| Individuals | 29780 (100.0) | 29559 (99.3) | 26433 (88.8) | 18925 (63.5) | 8378 (28.1) | 1886 (6.3) | 222 (0.7) | 9 (0.0) |
| GLP-1 receptor agonist | 221 (0.8) | 3126 (10.5) | 7508 (25.2) | 10547 (35.4) | 6492 (21.8) | 1664 (5.6) | 213 (0.7) | 9 (0.0) |
| Metformin | 26181 (87.9) | 2813 (9.4) | 304 (1.0) | 59 (0.2) | 11 (0.0) | <7 | <7 | <7 |
| Sulfonylureas | 4993 (16.8) | 10610 (35.6) | 3799 (12.8) | 882 (3.0) | 81 (0.3) | <7 | <7 | <7 |
| SGLT-2 inhibitors | 321 (1.1) | 2571 (8.6) | 2543 (8.5) | 2012 (6.8) | 672 (2.3) | 98 (0.3) | <7 | <7 |
| DPP-4 inhibitors | 613 (2.1) | 4996 (16.8) | 6108 (20.5) | 2665 (8.9) | 408 (1.4) | 43 (0.1) | <7 | <7 |
| Thiazolidinediones | 826 (2.8) | 4296 (14.4) | 3985 (13.4) | 815 (2.7) | 150 (0.5) | <7 | <7 | <7 |
| Insulin | <7 | 1042 (3.5) | 1890 (6.3) | 1853 (6.2) | 554 (1.9) | 81 (0.3) | <7 | <7 |
| Other glucose-lowering drugs | 200 (0.7) | 440 (1.5) | 480 (1.6) | 223 (0.7) | 46 (0.2) | <7 | <7 | <7 |
| **Individuals initiating GLP-1 receptor agonist therapy between 01 January 2007 and 31 December 2017** | | | | | | | | |
| Individuals | 18517 (100.0) | 18456 (99.7) | 16793 (90.7) | 11998 (64.8) | 4926 (26.6) | 953 (5.1) | <7 | <7 |
| GLP-1 receptor agonist | 61 (0.3) | 1663 (9.0) | 4795 (25.9) | 7072 (38.2) | 3973 (21.5) | 858 (4.6) | <7 | <7 |
| Metformin | 15946 (86.1) | 2147 (11.6) | 233 (1.3) | 44 (0.2) | 9 (0.0) | <7 | <7 | <7 |
| Sulfonylureas | 3514 (19.0) | 7572 (40.9) | 2492 (13.5) | 440 (2.4) | 24 (0.1) | <7 | <7 | <7 |
| SGLT-2 inhibitors | 14 (0.1) | 199 (1.1) | 400 (2.2) | 444 (2.4) | 202 (1.1) | 34 (0.2) | <7 | <7 |
| DPP-4 inhibitors | 185 (1.0) | 2414 (13.0) | 3600 (19.4) | 1839 (9.9) | 300 (1.6) | 34 (0.2) | <7 | <7 |
| Thiazolidinediones | 660 (3.6) | 3546 (19.1) | 3436 (18.6) | 633 (3.4) | 74 (0.4) | <7 | <7 | <7 |
| Insulin | <7 | 708 (3.8) | 1496 (8.1) | 1384 (7.5) | 330 (1.8) | 28 (0.2) | <7 | <7 |
| Other glucose-lowering drugs | 175 (0.9) | 400 (2.2) | 441 (2.4) | 207 (1.1) | 35 (0.2) | <7 | <7 | <7 |
| **Individuals initiating GLP-1 receptor agonist therapy between 01 January 2018 and 30 June 2023** | | | | | | | | |
| Individuals | 11263 (100.0) | 11103 (98.6) | 9640 (85.6) | 6927 (61.5) | 3452 (30.6) | 933 (8.3) | 127 (1.1) | 7 (0.1) |
| GLP-1 receptor agonist | 160 (1.4) | 1463 (13.0) | 2713 (24.1) | 3475 (30.8) | 2519 (22.4) | 806 (7.2) | 120 (1.1) | 7 (0.1) |
| Metformin | 10235 (90.9) | 666 (5.9) | 71 (0.6) | 15 (0.1) | <7 | <7 | <7 | <7 |
| Sulfonylureas | 1479 (13.1) | 3038 (27.0) | 1307 (11.6) | 442 (3.9) | 57 (0.5) | <7 | <7 | <7 |
| SGLT-2 inhibitors | 307 (2.7) | 2372 (21.1) | 2143 (19.0) | 1568 (13.9) | 470 (4.2) | 64 (0.6) | <7 | <7 |
| DPP-4 inhibitors | 428 (3.8) | 2582 (22.9) | 2508 (22.3) | 826 (7.3) | 108 (1.0) | 9 (0.1) | <7 | <7 |
| Thiazolidinediones | 166 (1.5) | 750 (6.7) | 549 (4.9) | 182 (1.6) | 76 (0.7) | <7 | <7 | <7 |
| Insulin | <7 | 334 (3.0) | 394 (3.5) | 469 (4.2) | 224 (2.0) | 53 (0.5) | <7 | <7 |
| Other glucose-lowering drugs | 25 (0.2) | 40 (0.4) | 39 (0.3) | 16 (0.1) | 11 (0.1) | <7 | <7 | <7 |
| Abbreviations: GLP-1 glucagon-like peptide-1, SGLT-2 sodium-glucose co-transporter 2, DPP-4 dipeptidyl peptidase-4. *First-line treatment was defined as the first non-insulin glucose-lowering therapy prescribed, while second-line to eighth-line treatments were defined as the initiation of any new glucose-lowering therapy, including insulin, not used in a previous treatment-line. | | | | | | | | |

| **Table S7. Glucose-lowering therapies (GLT)* used during the one year before and after glucagon-like peptide-1 (GLP-1) receptor agonist initiation, overall and stratified by initiation before 2018 and in 2018 or later.** | | | | |
| --- | --- | --- | --- | --- |
| **Use of GLT* within time intervals (days) respective to GLP-1 receptor agonist therapy initiation (day 0)** | **-361, -181** | **-180, 0** | **1, 181** | **182, 362** |
| **GLP-1 receptor agonist therapy initiation between 01-Jan-2007 and 30-Jun-2023** | | | | |
| Number of individuals assessed in each time interval | 29780 | 29780 | 20006 | 14910 |
| Metformin, n (%) | 25348 (85.1) | 26197 (88.0) | 17452 (87.2) | 12977 (87.0) |
| Sulfonylureas, n (%) | 14315 (48.1) | 15053 (50.5) | 8941 (44.7) | 6503 (43.6) |
| SGLT-2 inhibitors, n (%) | 5850 (19.6) | 6828 (22.9) | 3259 (16.3) | 2479 (16.6) |
| DPP-4 inhibitors, n (%) | 10903 (36.6) | 12019 (40.4) | 1851 (9.3) | 658 (4.4) |
| Thiazolidinediones, n (%) | 4714 (15.8) | 4411 (14.8) | 1489 (7.4) | 982 (6.6) |
| Other GLT, n (%) | 359 (1.2) | 354 (1.2) | 127 (0.6) | 86 (0.6) |
| Insulin, n (%) | 4378 (14.7) | 4878 (16.4) | 2911 (14.6) | 2319 (15.6) |
| Number of co-prescribed GLT |  | | | |
| GLT=0, n (%) | 1305 (4.4) | 349 (1.2) | 607 (3.0) | 503 (3.4) |
| GLT=1, n (%) | 4885 (16.4) | 4329 (14.5) | 6156 (30.8) | 4865 (32.6) |
| GLT=2, n (%) | 11566 (38.8) | 11912 (40.0) | 10096 (50.5) | 7637 (51.2) |
| GLT=3, n (%) | 10310 (34.6) | 11253 (37.8) | 2915 (14.6) | 1759 (11.8) |
| GLT≥3, n (%) | 12024 (40.4) | 13190 (44.3) | 3147 (15.7) | 1905 (12.8) |
| GLT≥4, n (%) | 1714 (5.8) | 1937 (6.5) | 232 (1.2) | 146 (1.0) |
| **GLP-1 receptor agonist therapy initiation between 01-Jan-2007 and 31-Dec-2017** | | | | |
| Number of individuals assessed in each time interval | 18517 | 18517 | 13171 | 10115 |
| Metformin, n (%) | 16202 (87.5) | 16662 (90.0) | 11774 (89.4) | 9038 (89.4) |
| Sulfonylureas, n (%) | 10185 (55.0) | 10704 (57.8) | 6666 (50.6) | 4982 (49.3) |
| SGLT-2 inhibitors, n (%) | 801 (4.3) | 1105 (6.0) | 513 (3.9) | 535 (5.3) |
| DPP-4 inhibitors, n (%) | 6262 (33.8) | 7117 (38.4) | 1117 (8.5) | 389 (3.8) |
| Thiazolidinediones, n (%) | 4208 (22.7) | 3911 (21.1) | 1305 (9.9) | 858 (8.5) |
| Other GLT, n (%) | 331 (1.8) | 327 (1.8) | 114 (0.9) | 78 (0.8) |
| Insulin, n (%) | 3288 (17.8) | 3574 (19.3) | 2168 (16.5) | 1781 (17.6) |
| Number of co-prescribed GLT |  | | | |
| GLT=0, n (%) | 560 (3.0) | 145 (0.8) | 269 (2.0) | 237 (2.3) |
| GLT=1, n (%) | 2817 (15.2) | 2375 (12.8) | 4049 (30.7) | 3299 (32.6) |
| GLT=2, n (%) | 7815 (42.2) | 7946 (42.9) | 7071 (53.7) | 5463 (54.0) |
| GLT=3, n (%) | 6503 (35.1) | 7115 (38.4) | 1665 (12.6) | 1030 (10.2) |
| GLT≥3, n (%) | 7325 (39.6) | 8051 (43.5) | 1782 (13.5) | 1116 (11.0) |
| GLT≥4, n (%) | 822 (4.4) | 936 (5.1) | 117 (0.9) | 86 (0.9) |
| **GLP-1 receptor agonist therapy initiation between 01-Jan-2018 and 30-Jun-2023** | | | | |
| Number of individuals assessed in each time interval | 11263 | 11263 | 6835 | 4795 |
| Metformin, n (%) | 9146 (81.2) | 9535 (84.7) | 5678 (83.1) | 3939 (82.1) |
| Sulfonylureas, n (%) | 4130 (36.7) | 4349 (38.6) | 2275 (33.3) | 1521 (31.7) |
| SGLT-2 inhibitors, n (%) | 5049 (44.8) | 5723 (50.8) | 2746 (40.2) | 1944 (40.5) |
| DPP-4 inhibitors, n (%) | 4641 (41.2) | 4902 (43.5) | 734 (10.7) | 269 (5.6) |
| Thiazolidinediones, n (%) | 506 (4.5) | 500 (4.4) | 184 (2.7) | 124 (2.6) |
| Other GLT, n (%) | 28 (0.2) | 27 (0.2) | 13 (0.2) | 8 (0.2) |
| Insulin, n (%) | 1090 (9.7) | 1304 (11.6) | 743 (10.9) | 538 (11.2) |
| Number of co-prescribed GLT |  | | | |
| GLT=0, n (%) | 745 (6.6) | 204 (1.8) | 338 (4.9) | 266 (5.5) |
| GLT=1, n (%) | 2068 (18.4) | 1954 (17.3) | 2107 (30.8) | 1566 (32.7) |
| GLT=2, n (%) | 3751 (33.3) | 3966 (35.2) | 3025 (44.3) | 2174 (45.3) |
| GLT=3, n (%) | 3807 (33.8) | 4138 (36.7) | 1250 (18.3) | 729 (15.2) |
| GLT≥3, n (%) | 4699 (41.7) | 5139 (45.6) | 1365 (20.0) | 789 (16.5) |
| GLT≥4, n (%) | 892 (7.9) | 1001 (8.9) | 115 (1.7) | 60 (1.3) |
| Abbreviations: GLT glucose-lowering therapies, GLP-1 glucagon-like peptide-1, SGLT-2 sodium-glucose co-transporter 2, DPP-4 dipeptidyl peptidase-4. *Defined as having at least one prescription for GLT specified in Supplementary Table S2 within the specified time interval, while adhering to GLP-1 receptor agonist therapy upon initiation. | | | | |

| **Table S8. Characteristics of individuals initiating glucagon-like peptide-1 (GLP-1) receptor agonist therapy between 01 January 2018 and 30 June 2023, stratified by cardiovascular disease (CVD) history.** | | | |
| --- | --- | --- | --- |
| **Characteristics** | **Initiation post-2018** | **CVD history** | **No CVD history** |
| Individuals included, n (%) | 11263 (100.0) | 2103 (18.7) | 9160 (81.3) |
| Age at initiation, years, median (IQR) | 60.8 [53.1, 68.6] | 66.1 [59.1, 72.9] | 59.4 [51.9, 67.1] |
| Female sex assigned at birth, n (%) | 5034 (44.7) | 716 (34.0) | 4318 (47.1) |
| Type 2 diabetes duration, years, median (IQR) | 6.7 [3.2, 11.5] | 7.8 [3.9, 13.2] | 6.5 [3.1, 11.1] |
| **GLP-1 receptor agonist agent initiated, n (%)** | | | |
| Semaglutide | 5084 (45.1) | 906 (43.1) | 4178 (45.6) |
| Dulaglutide | 4662 (41.4) | 886 (42.1) | 3776 (41.2) |
| Liraglutide | 1234 (11.0) | 260 (12.4) | 974 (10.6) |
| Exenatide | 241 (2.1) | 46 (2.2) | 195 (2.1) |
| Lixisenatide | 42 (0.4) | <7 | 37 (0.4) |
| **Previous use of glucose-lowering therapies*, n (%)** | | | |
| Metformin | 10932 (97.1) | 2036 (96.8) | 8896 (97.1) |
| Sulfonylureas | 6286 (55.8) | 1347 (64.1) | 4939 (53.9) |
| SGLT-2 inhibitors | 6827 (60.6) | 1191 (56.6) | 5636 (61.5) |
| DPP-4 inhibitors | 6456 (57.3) | 1248 (59.3) | 5208 (56.9) |
| Thiazolidinediones | 1725 (15.3) | 364 (17.3) | 1361 (14.9) |
| Insulin | 1448 (12.9) | 398 (18.9) | 1050 (11.5) |
| Other glucose-lowering drugs | 132 (1.2) | 41 (1.9) | 91 (1.0) |
| **Concurrent use of glucose-lowering therapies†, n (%)** | | | |
| Metformin | 9535 (84.7) | 1709 (81.3) | 7826 (85.4) |
| Sulfonylureas | 4349 (38.6) | 911 (43.3) | 3438 (37.5) |
| SGLT-2 inhibitors | 5723 (50.8) | 1006 (47.8) | 4717 (51.5) |
| DPP-4 inhibitors | 4902 (43.5) | 941 (44.7) | 3961 (43.2) |
| Thiazolidinediones | 500 (4.4) | 61 (2.9) | 439 (4.8) |
| Insulin | 1304 (11.6) | 373 (17.7) | 931 (10.2) |
| Other glucose-lowering drugs | 27 (0.2) | 10 (0.5) | 17 (0.2) |
| **Comorbidities‡, n (%)** | | | |
| Cardiovascular disease | 2103 (18.7) | 2103 (100.0) | <7 |
| Hypertension | 7174 (63.7) | 1535 (73.0) | 5639 (61.6) |
| Dyslipidemia | 2189 (19.4) | 601 (28.6) | 1588 (17.3) |
| Depression | 4620 (41.0) | 846 (40.2) | 3774 (41.2) |
| Asthma | 2540 (22.6) | 466 (22.2) | 2074 (22.6) |
| Chronic obstructive pulmonary disease | 2891 (25.7) | 615 (29.2) | 2276 (24.8) |
| Chronic kidney disease | 1774 (15.8) | 566 (26.9) | 1208 (13.2) |
| **Laboratory and vital sign measurements§** | | | |
| BMI, kg/m^2^, median (IQR) | 34.0 [30.5, 38.0] | 33.2 [30.1, 37.1] | 34.1 [30.6, 38.2] |
| BMI ≥35, n (%) | 3654 (32.4) | 628 (29.9) | 3026 (33.0) |
| BMI missing, n (%) | 2696 (23.9) | 497 (23.6) | 2199 (24.0) |
| eGFR, ml/min per 1.72m^2^, median (IQR) | 98.20 [82.00, 107.70] | 88.4 [63.9, 99.4] | 100.0 [86.6, 109.1] |
| eGFR <60, n (%) | 1075 (9.5) | 436 (20.7) | 639 (7.0) |
| Missing eGFR, n (%) | 334 (3.0) | 54 (2.6) | 280 (3.1) |
| HbA1c, %, median (IQR) | 8.19 [7.64, 8.55] | 8.19 [7.64, 8.65] | 8.19 [7.64, 8.55] |
| **Line of glucose-lowering therapy at GLP-1 receptor agonist initiation, n (%)** | | | |
| First-line | 160 (1.4) | 23 (1.1) | 137 (1.5) |
| Second-line | 1463 (13.0) | 206 (9.8) | 1257 (13.7) |
| Third-line | 2713 (24.1) | 497 (23.6) | 2216 (24.2) |
| Fourth-line | 3475 (30.8) | 654 (31.1) | 2821 (30.8) |
| Fifth-line | 2519 (22.4) | 504 (24.0) | 2015 (22.0) |
| Sixth to eighth-line | 933 (8.3) | 219 (10.4) | 714 (7.8) |
| Abbreviations: GLP-1 glucagon-like peptide-1, SGLT-2 sodium-glucose co-transporter 2, DPP-4 dipeptidyl peptidase-4, HbA1c glycated hemoglobin, BMI body-mass index, eGFR estimated glomerular filtration rate.  *Defined as having at least one prescription for a glucose-lowering therapy specified in Supplementary Table S2 before GLP-1 receptor agonist initiation. †Defined as having at least one prescription for a glucose-lowering therapy specified in Supplementary Table S2 within the 180 days before or at GLP-1 receptor agonist initiation. ‡Diagnosis recorded before or at GLP-1 receptor agonist initiation. §Most recent measurement recorded within the year before or at GLP-1 receptor agonist initiation. | | | |

| **Table S9. Glucose-lowering therapies (GLT)* used during the one year before and after glucagon-like peptide-1 (GLP-1) receptor agonist initiation in 2018 or later, by subgroups of cardiovascular disease (CVD) history at initiation.** | | | | |
| --- | --- | --- | --- | --- |
| **Use of GLT* within time intervals (days) respective to GLP-1 receptor agonist therapy initiation (day 0)** | **-361, -181** | **-180, 0** | **1, 181** | **182, 362** |
| **GLP-1 receptor agonist therapy initiation between 01-Jan-2018 and 30-Jun-2023 with a CVD history** | | | | |
| Individuals with a CVD history, n (%) | 2103 (100.0) | 2103 (100.0) | 1265 (100.0) | 901 (100.0) |
| Metformin, n (%) | 1674 (79.6) | 1709 (81.3) | 1006 (79.5) | 711 (78.9) |
| Sulfonylureas, n (%) | 880 (41.8) | 911 (43.3) | 499 (39.4) | 336 (37.3) |
| SGLT-2 inhibitors, n (%) | 875 (41.6) | 1006 (47.8) | 472 (37.3) | 340 (37.7) |
| DPP-4 inhibitors, n (%) | 896 (42.6) | 941 (44.7) | 152 (12.0) | 60 (6.7) |
| Thiazolidinediones, n (%) | 62 (2.9) | 61 (2.9) | 20 (1.6) | 11 (1.2) |
| Other GLT, n (%) | 11 (0.5) | 10 (0.5) | <7 | <7 |
| Insulin, n (%) | 320 (15.2) | 373 (17.7) | 221 (17.5) | 157 (17.4) |
| Number of co-prescribed GLT |  | | | |
| GLT=0, n (%) | 89 (4.2) | 33 (1.6) | 57 (4.5) | 42 (4.7) |
| GLT=1, n (%) | 366 (17.4) | 325 (15.5) | 339 (26.8) | 273 (30.3) |
| GLT=2, n (%) | 752 (35.8) | 759 (36.1) | 596 (47.1) | 425 (47.2) |
| GLT=3, n (%) | 742 (35.3) | 781 (37.1) | 247 (19.5) | 148 (16.4) |
| GLT≥3, n (%) | 896 (42.6) | 986 (46.9) | 273 (21.6) | 161 (17.9) |
| GLT≥4, n (%) | 154 (7.3) | 205 (9.7) | 26 (2.1) | 13 (1.4) |
| **GLP-1 receptor agonist therapy initiation between 01-Jan-2018 and 30-Jun-2023 without a CVD history** | | | | |
| Individuals without a CVD history, n (%) | 9160 (100.0) | 9160 (100.0) | 5570 (100.0) | 3894 (100.0) |
| Metformin, n (%) | 7472 (81.6) | 7826 (85.4) | 4672 (83.9) | 3228 (82.9) |
| Sulfonylureas, n (%) | 3250 (35.5) | 3438 (37.5) | 1776 (31.9) | 1185 (30.4) |
| SGLT-2 inhibitors, n (%) | 4174 (45.6) | 4717 (51.5) | 2274 (40.8) | 1604 (41.2) |
| DPP-4 inhibitors, n (%) | 3745 (40.9) | 3961 (43.2) | 582 (10.4) | 209 (5.4) |
| Thiazolidinediones, n (%) | 444 (4.8) | 439 (4.8) | 164 (2.9) | 113 (2.9) |
| Other GLT, n (%) | 17 (0.2) | 17 (0.2) | 7 (0.1) | <7 |
| Insulin, n (%) | 770 (8.4) | 931 (10.2) | 522 (9.4) | 381 (9.8) |
| Number of co-prescribed GLT |  | | | |
| GLT=0, n (%) | 656 (7.2) | 171 (1.9) | 281 (5.0) | 224 (5.8) |
| GLT=1, n (%) | 1702 (18.6) | 1629 (17.8) | 1768 (31.7) | 1293 (33.2) |
| GLT=2, n (%) | 2999 (32.7) | 3207 (35.0) | 2429 (43.6) | 1749 (44.9) |
| GLT=3, n (%) | 3065 (33.5) | 3357 (36.6) | 1003 (18.0) | 581 (14.9) |
| GLT≥3, n (%) | 3803 (41.5) | 4153 (45.3) | 1092 (19.6) | 628 (16.1) |
| GLT≥4, n (%) | 738 (8.1) | 796 (8.7) | 89 (1.6) | 47 (1.2) |
| Abbreviations: GLT glucose-lowering therapies, GLP-1 glucagon-like peptide-1, CVD cardiovascular disease, SGLT-2 sodium-glucose co-transporter 2, DPP-4 dipeptidyl peptidase-4. *Defined as having at least one prescription for GLT specified in Supplementary Table S2 within the specified time interval, while adhering to GLP-1 receptor agonist therapy upon initiation. | | | | |

| **Table S10. Glucose-lowering therapies from the first prescription up to glucagon-like peptide-1 (GLP-1) receptor agonist initiation between 01 January 2018 and 30 June 2023, stratified by treatment line* and cardiovascular disease (CVD) history at GLP-1 receptor agonist initiation.** | | | | | | | | |
| --- | --- | --- | --- | --- | --- | --- | --- | --- |
| **Glucose-lowering therapies among individuals with a CVD history,**  **n (%)** | **First-line** | **Second-line** | **Third-line** | **Fourth-line** | **Fifth-line** | **Sixth-line** | **Seventh-line** | **Eighth-line** |
| Individuals | 2103 (100.0) | 2080 (98.9) | 1874 (89.1) | 1377 (65.5) | 723 (34.4) | 219 (10.4) | 38 (1.8) | <7 |
| GLP-1 receptor agonist | 23 (1.1) | 206 (9.8) | 497 (23.6) | 654 (31.1) | 504 (24.0) | 181 (8.6) | 35 (1.7) | <7 |
| Metformin | 1837 (87.4) | 177 (8.4) | 21 (1.0) | <7 | <7 | <7 | <7 | <7 |
| Sulfonylureas | 330 (15.7) | 673 (32.0) | 262 (12.5) | 76 (3.6) | 12 (0.6) | <7 | <7 | <7 |
| SGLT-2 inhibitors | 52 (2.5) | 384 (18.3) | 349 (16.6) | 300 (14.3) | 107 (5.1) | 19 (0.9) | <7 | <7 |
| DPP-4 inhibitors | 101 (4.8) | 433 (20.6) | 495 (23.5) | 183 (8.7) | 32 (1.5) | <7 | <7 | <7 |
| Thiazolidinediones | 34 (1.6) | 145 (6.9) | 142 (6.8) | 32 (1.5) | 11 (0.5) | <7 | <7 | <7 |
| Insulin | <7 | 81 (3.9) | 116 (5.5) | 133 (6.3) | 58 (2.8) | 15 (0.7) | <7 | <7 |
| Other glucose-lowering drugs | 9 (0.4) | 11 (0.5) | 12 (0.6) | <7 | <7 | <7 | <7 | <7 |
| **Glucose-lowering therapies among individuals without a CVD history, n (%)** | **First-line** | **Second-line** | **Third-line** | **Fourth-line** | **Fifth-line** | **Sixth-line** | **Seventh-line** | **Eighth-line** |
| Individuals | 9160 (100.0) | 9023 (98.5) | 7766 (84.8) | 5550 (60.6) | 2729 (29.8) | 714 (7.8) | 89 (1.0) | <7 |
| GLP-1 receptor agonist | 137 (1.5) | 1257 (13.7) | 2216 (24.2) | 2821 (30.8) | 2015 (22.0) | 625 (6.8) | 85 (0.9) | <7 |
| Metformin | 8398 (91.7) | 489 (5.3) | 50 (0.5) | 9 (0.1) | <7 | <7 | <7 | <7 |
| Sulfonylureas | 1149 (12.5) | 2365 (25.8) | 1045 (11.4) | 366 (4.0) | 45 (0.5) | <7 | <7 | <7 |
| SGLT-2 inhibitors | 255 (2.8) | 1988 (21.7) | 1794 (19.6) | 1268 (13.8) | 363 (4.0) | 45 (0.5) | <7 | <7 |
| DPP-4 inhibitors | 327 (3.6) | 2149 (23.5) | 2013 (22.0) | 643 (7.0) | 76 (0.8) | <7 | <7 | <7 |
| Thiazolidinediones | 132 (1.4) | 605 (6.6) | 407 (4.4) | 150 (1.6) | 65 (0.7) | <7 | <7 | <7 |
| Insulin | <7 | 253 (2.8) | 278 (3.0) | 336 (3.7) | 166 (1.8) | 38 (0.4) | <7 | <7 |
| Other glucose-lowering drugs | 16 (0.2) | 29 (0.3) | 27 (0.3) | 11 (0.1) | 7 (0.1) | <7 | <7 | <7 |
| Abbreviations: GLP-1 glucagon-like peptide-1, SGLT-2 sodium-glucose co-transporter 2, DPP-4 dipeptidyl peptidase-4. *First-line treatment was defined as the first non-insulin glucose-lowering therapy prescribed, while second-line to eighth-line treatments were defined as the initiation of any new glucose-lowering therapy, including insulin, not used in a previous treatment-line. | | | | | | | | |

| **Table S11. Characteristics of individuals initiating glucagon-like peptide-1 (GLP-1) receptor agonist therapy between 01 January 2018 and 30 June 2023, stratified by body-mass index (BMI)§ at initiation.** | | | |
| --- | --- | --- | --- |
| **Characteristics** | **BMI ≥35 kg/m^2^** | **BMI <35 kg/m^2^** | **BMI missing** |
| Individuals included, n (%) | 3654 (32.4) | 4913 (43.7) | 2696 (23.9) |
| Age at initiation, years, median (IQR) | 59.0 [51.6, 66.7] | 62.9 [55.6, 70.5] | 58.8 [50.9, 67.0] |
| Female sex assigned at birth, n (%) | 1789 (49.0) | 1861 (37.9) | 1384 (51.3) |
| Type 2 diabetes duration, years, median (IQR) | 5.3 [2.5, 9.6] | 8.2 [4.4, 12.7] | 5.90 [2.7, 11.1] |
| **GLP-1 receptor agonist agent initiated, n (%)** | | | |
| Semaglutide | 1604 (43.9) | 2243 (45.7) | 1237 (45.9) |
| Dulaglutide | 1502 (41.1) | 2070 (42.1) | 1090 (40.4) |
| Liraglutide | 440 (12.0) | 477 (9.7) | 317 (11.8) |
| Exenatide | 94 (2.6) | 104 (2.1) | 43 (1.6) |
| Lixisenatide | 14 (0.4) | 19 (0.4) | 9 (0.3) |
| **Previous use of glucose-lowering therapies*, n (%)** | | | |
| Metformin | 3533 (96.7) | 4831 (98.3) | 2568 (95.3) |
| Sulfonylureas | 1735 (47.5) | 3196 (65.1) | 1355 (50.3) |
| SGLT-2 inhibitors | 2067 (56.6) | 3305 (67.3) | 1455 (54.0) |
| DPP-4 inhibitors | 1860 (50.9) | 3238 (65.9) | 1358 (50.4) |
| Thiazolidinediones | 462 (12.6) | 891 (18.1) | 372 (13.8) |
| Insulin | 397 (10.9) | 641 (13.0) | 410 (15.2) |
| Other glucose-lowering drugs | 31 (0.8) | 73 (1.5) | 28 (1.0) |
| **Concurrent use of glucose-lowering therapies†, n (%)** | | | |
| Metformin | 3080 (84.3) | 4222 (85.9) | 2233 (82.8) |
| Sulfonylureas | 1227 (33.6) | 2214 (45.1) | 908 (33.7) |
| SGLT-2 inhibitors | 1706 (46.7) | 2784 (56.7) | 1233 (45.7) |
| DPP-4 inhibitors | 1436 (39.3) | 2456 (50.0) | 1010 (37.5) |
| Thiazolidinediones | 167 (4.6) | 242 (4.9) | 91 (3.4) |
| Insulin | 367 (10.0) | 554 (11.3) | 383 (14.2) |
| Other glucose-lowering drugs | <7 | 15 (0.3) | <7 |
| **Comorbidities‡, n (%)** | | | |
| Cardiovascular disease | 628 (17.2) | 978 (19.9) | 497 (18.4) |
| Hypertension | 2402 (65.7) | 3075 (62.6) | 1697 (62.9) |
| Dyslipidemia | 673 (18.4) | 1053 (21.4) | 463 (17.2) |
| Depression | 1610 (44.1) | 1804 (36.7) | 1206 (44.7) |
| Asthma | 900 (24.6) | 924 (18.8) | 716 (26.6) |
| Chronic obstructive pulmonary disease | 1021 (27.9) | 1073 (21.8) | 797 (29.6) |
| Chronic kidney disease | 529 (14.5) | 862 (17.5) | 383 (14.2) |
| **Laboratory and vital sign measurements§** | | | |
| BMI, kg/m^2^, median (IQR) | 38.7 [36.7, 41.8] | 31.0 [28.8, 32.9] | NA [NA, NA] |
| BMI ≥35, n (%) | 3654 (100.0) | <7 | <7 |
| BMI missing, n (%) | <7 | <7 | 2696 (100.0) |
| eGFR, ml/min per 1.72m^2^, median (IQR) | 100.0 [85.3, 108.7] | 95.9 [78.2, 105.0] | 100.4 [85.7, 110.5] |
| eGFR <60, n (%) | 289 (7.9) | 547 (11.1) | 239 (8.9) |
| Missing eGFR, n (%) | 63 (1.7) | 96 (2.0) | 175 (6.5) |
| HbA1c, %, median (IQR) | 8.10 [7.46, 8.55] | 8.28 [7.82, 8.65] | 8.10 [7.46, 8.55] |
| **Line of glucose-lowering therapy at GLP-1 receptor agonist initiation, n (%)** | | | |
| First-line | 61 (1.7) | 19 (0.4) | 80 (3.0) |
| Second-line | 628 (17.2) | 371 (7.6) | 464 (17.2) |
| Third-line | 1000 (27.4) | 1037 (21.1) | 676 (25.1) |
| Fourth-line | 1101 (30.1) | 1601 (32.6) | 773 (28.7) |
| Fifth-line | 644 (17.6) | 1369 (27.9) | 506 (18.8) |
| Sixth to eighth-line | 220 (6.0) | 516 (10.5) | 197 (7.3) |
| Abbreviations: GLP-1 glucagon-like peptide-1, SGLT-2 sodium-glucose co-transporter 2, DPP-4 dipeptidyl peptidase-4, HbA1c glycated hemoglobin, BMI body-mass index, eGFR estimated glomerular filtration rate.  *Defined as having at least one prescription for a glucose-lowering therapy specified in Supplementary Table S2 before GLP-1 receptor agonist initiation. †Defined as having at least one prescription for a glucose-lowering therapy specified in Supplementary Table S2 within the 180 days before or at GLP-1 receptor agonist initiation. ‡Diagnosis recorded before or at GLP-1 receptor agonist initiation. §Most recent measurement recorded within the year before or at GLP-1 receptor agonist initiation. | | | |

| **Table S12. Glucose-lowering therapies (GLT)* used during the one year before and after glucagon-like peptide-1 (GLP-1) receptor agonist initiation in 2018 or later, stratified by body-mass index (BMI)† category at initiation.** | | | | |
| --- | --- | --- | --- | --- |
| **Use of GLT* within time intervals (days) respective to GLP-1 receptor agonist therapy initiation (day 0)** | **-361, -181** | **-180, 0** | **1, 181** | **182, 362** |
| **GLP-1 receptor agonist therapy initiation between 01-Jan-2018 and 30-Jun-2023 and BMI ≥35 kg/m^2^** | | | | |
| Individuals with BMI ≥35 kg/m^2^, n (%) | 3654 (100.0) | 3654 (100.0) | 2344 (100.0) | 1683 (100.0) |
| Metformin, n (%) | 2920 (79.9) | 3080 (84.3) | 1930 (82.3) | 1368 (81.3) |
| Sulfonylureas, n (%) | 1126 (30.8) | 1227 (33.6) | 662 (28.2) | 452 (26.9) |
| SGLT-2 inhibitors, n (%) | 1441 (39.4) | 1706 (46.7) | 840 (35.8) | 624 (37.1) |
| DPP-4 inhibitors, n (%) | 1340 (36.7) | 1436 (39.3) | 231 (9.9) | 88 (5.2) |
| Thiazolidinediones, n (%) | 163 (4.5) | 167 (4.6) | 63 (2.7) | 46 (2.7) |
| Other GLT, n (%) | <7 | <7 | <7 | <7 |
| Insulin, n (%) | 302 (8.3) | 367 (10.0) | 228 (9.7) | 176 (10.5) |
| Number of co-prescribed GLT |  | | | |
| GLT=0, n (%) | 296 (8.1) | 81 (2.2) | 138 (5.9) | 112 (6.7) |
| GLT=1, n (%) | 840 (23.0) | 776 (21.2) | 847 (36.1) | 629 (37.4) |
| GLT=2, n (%) | 1279 (35.0) | 1410 (38.6) | 988 (42.2) | 715 (42.5) |
| GLT=3, n (%) | 1065 (29.1) | 1164 (31.9) | 351 (15.0) | 211 (12.5) |
| GLT≥3, n (%) | 1239 (33.9) | 1387 (38.0) | 371 (15.8) | 227 (13.5) |
| GLT≥4, n (%) | 174 (4.8) | 223 (6.1) | 20 (0.9) | 16 (1.0) |
| **GLP-1 receptor agonist therapy initiation between 01-Jan-2018 and 30-Jun-2023 and BMI <35 kg/m^2^** | | | | |
| Individuals with BMI <35 kg/m^2^, n (%) | 4913 (100.0) | 4913 (100.0) | 2862 (100.0) | 1992 (100.0) |
| Metformin, n (%) | 4171 (84.9) | 4222 (85.9) | 2410 (84.2) | 1676 (84.1) |
| Sulfonylureas, n (%) | 2162 (44.0) | 2214 (45.1) | 1122 (39.2) | 747 (37.5) |
| SGLT-2 inhibitors, n (%) | 2546 (51.8) | 2784 (56.7) | 1297 (45.3) | 886 (44.5) |
| DPP-4 inhibitors, n (%) | 2363 (48.1) | 2456 (50.0) | 336 (11.7) | 120 (6.0) |
| Thiazolidinediones, n (%) | 254 (5.2) | 242 (4.9) | 86 (3.0) | 52 (2.6) |
| Other GLT, n (%) | 17 (0.3) | 15 (0.3) | 7 (0.2) | <7 |
| Insulin, n (%) | 466 (9.5) | 554 (11.3) | 297 (10.4) | 217 (10.9) |
| Number of co-prescribed GLT |  | | | |
| GLT=0, n (%) | 162 (3.3) | 43 (0.9) | 108 (3.8) | 80 (4.0) |
| GLT=1, n (%) | 632 (12.9) | 558 (11.4) | 718 (25.1) | 554 (27.8) |
| GLT=2, n (%) | 1577 (32.1) | 1603 (32.6) | 1340 (46.8) | 958 (48.1) |
| GLT=3, n (%) | 1993 (40.6) | 2130 (43.4) | 629 (22.0) | 369 (18.5) |
| GLT≥3, n (%) | 2542 (51.7) | 2709 (55.1) | 696 (24.3) | 400 (20.1) |
| GLT≥4, n (%) | 549 (11.2) | 579 (11.8) | 67 (2.3) | 31 (1.6) |
| **GLP-1 receptor agonist therapy initiation between 01-Jan-2018 and 30-Jun-2023 without a recent BMI measurement recorded** | | | | |
| Individuals without a recent BMI measurement, n (%) | 2696 (100.0) | 2696 (100.0) | 1629 (100.0) | 1120 (100.0) |
| Metformin, n (%) | 2055 (76.2) | 2233 (82.8) | 1338 (82.1) | 895 (79.9) |
| Sulfonylureas, n (%) | 842 (31.2) | 908 (33.7) | 491 (30.1) | 322 (28.7) |
| SGLT-2 inhibitors, n (%) | 1062 (39.4) | 1233 (45.7) | 609 (37.4) | 434 (38.8) |
| DPP-4 inhibitors, n (%) | 938 (34.8) | 1010 (37.5) | 167 (10.3) | 61 (5.4) |
| Thiazolidinediones, n (%) | 89 (3.3) | 91 (3.4) | 35 (2.1) | 26 (2.3) |
| Other GLT, n (%) | 7 (0.3) | <7 | <7 | <7 |
| Insulin, n (%) | 322 (11.9) | 383 (14.2) | 218 (13.4) | 145 (12.9) |
| Number of co-prescribed GLT |  | | | |
| GLT=0, n (%) | 287 (10.6) | 80 (3.0) | 92 (5.6) | 74 (6.6) |
| GLT=1, n (%) | 596 (22.1) | 620 (23.0) | 542 (33.3) | 383 (34.2) |
| GLT=2, n (%) | 895 (33.2) | 953 (35.3) | 697 (42.8) | 501 (44.7) |
| GLT=3, n (%) | 749 (27.8) | 844 (31.3) | 270 (16.6) | 149 (13.3) |
| GLT≥3, n (%) | 918 (34.1) | 1043 (38.7) | 298 (18.3) | 162 (14.5) |
| GLT≥4, n (%) | 169 (6.3) | 199 (7.4) | 28 (1.7) | 13 (1.2) |
| Abbreviations: GLT glucose-lowering therapies, GLP-1 glucagon-like peptide-1, BMI body-mass index, SGLT-2 sodium-glucose co-transporter 2, DPP-4 dipeptidyl peptidase-4. *Defined as having at least one prescription for GLT specified in Supplementary Table S2 within the specified time interval, while adhering to GLP-1 receptor agonist therapy upon initiation. †Most recent measurement recorded within the year before or at GLP-1 receptor agonist initiation. | | | | |

| **Table S13. Glucose-lowering therapies from the first prescription up to glucagon-like peptide-1 (GLP-1) receptor agonist initiation between 01 January 2018 and 30 June 2023, stratified by treatment line* and by body-mass index (BMI)† category at initiation.** | | | | | | | | |
| --- | --- | --- | --- | --- | --- | --- | --- | --- |
| **Glucose-lowering therapies among individuals with BMI ≥35 kg/m^2^,**  **n (%)** | **First-line** | **Second-line** | **Third-line** | **Fourth-line** | **Fifth-line** | **Sixth-line** | **Seventh-line** | **Eighth-line** |
| Individuals | 3654 (100.0) | 3593 (98.3) | 2965 (81.1) | 1965 (53.8) | 864 (23.6) | 220 (6.0) | 21 (0.6) | <7 |
| GLP-1 receptor agonist | 61 (1.7) | 628 (17.2) | 1000 (27.4) | 1101 (30.1) | 644 (17.6) | 199 (5.4) | 19 (0.5) | <7 |
| Metformin | 3395 (92.9) | 140 (3.8) | 13 (0.4) | <7 | <7 | <7 | <7 | <7 |
| Sulfonylureas | 362 (9.9) | 849 (23.2) | 393 (10.8) | 130 (3.6) | 12 (0.3) | <7 | <7 | <7 |
| SGLT-2 inhibitors | 108 (3.0) | 883 (24.2) | 653 (17.9) | 347 (9.5) | 94 (2.6) | 8 (0.2) | <7 | <7 |
| DPP-4 inhibitors | 120 (3.3) | 819 (22.4) | 667 (18.3) | 229 (6.3) | 24 (0.7) | <7 | <7 | <7 |
| Thiazolidinediones | 40 (1.1) | 210 (5.7) | 137 (3.7) | 46 (1.3) | 28 (0.8) | <7 | <7 | <7 |
| Insulin | <7 | 97 (2.7) | 115 (3.1) | 124 (3.4) | 61 (1.7) | 10 (0.3) | <7 | <7 |
| Other glucose-lowering drugs | <7 | <7 | 13 (0.4) | <7 | <7 | <7 | <7 | <7 |
| **Glucose-lowering therapies among individuals with BMI <35 kg/m^2^,**  **n (%)** | **First-line** | **Second-line** | **Third-line** | **Fourth-line** | **Fifth-line** | **Sixth-line** | **Seventh-line** | **Eighth-line** |
| Individuals | 4913 (100.0) | 4894 (99.6) | 4523 (92.1) | 3486 (71.0) | 1885 (38.4) | 516 (10.5) | 72 (1.5) | <7 |
| GLP-1 receptor agonist | 19 (0.4) | 371 (7.6) | 1037 (21.1) | 1601 (32.6) | 1369 (27.9) | 444 (9.0) | 68 (1.4) | <7 |
| Metformin | 4418 (89.9) | 372 (7.6) | 43 (0.9) | 8 (0.2) | <7 | <7 | <7 | <7 |
| Sulfonylureas | 771 (15.7) | 1527 (31.1) | 645 (13.1) | 234 (4.8) | 33 (0.7) | 0 (0.0) | <7 | <7 |
| SGLT-2 inhibitors | 129 (2.6) | 939 (19.1) | 1045 (21.3) | 926 (18.8) | 268 (5.5) | 39 (0.8) | <7 | <7 |
| DPP-4 inhibitors | 204 (4.2) | 1220 (24.8) | 1323 (26.9) | 432 (8.8) | 57 (1.2) | <7 | <7 | <7 |
| Thiazolidinediones | 95 (1.9) | 371 (7.6) | 285 (5.8) | 100 (2.0) | 39 (0.8) | <7 | <7 | <7 |
| Insulin | <7 | 130 (2.6) | 155 (3.2) | 211 (4.3) | 117 (2.4) | 30 (0.6) | <7 | <7 |
| Other glucose-lowering drugs | 14 (0.3) | 23 (0.5) | 20 (0.4) | 8 (0.2) | 7 (0.1) | <7 | <7 | <7 |
| **Glucose-lowering therapies among individuals without a recent BMI measurement recorded, n (%)** | **First-line** | **Second-line** | **Third-line** | **Fourth-line** | **Fifth-line** | **Sixth-line** | **Seventh-line** | **Eighth-line** |
| Individuals | 2696 (100.0) | 2616 (97.0) | 2152 (79.8) | 1476 (54.7) | 703 (26.1) | 197 (7.3) | 34 (1.3) | <7 |
| GLP-1 receptor agonist | 80 (3.0) | 464 (17.2) | 676 (25.1) | 773 (28.7) | 506 (18.8) | 163 (6.0) | 33 (1.2) | <7 |
| Metformin | 2422 (89.8) | 154 (5.7) | 15 (0.6) | <7 | <7 | <7 | <7 | <7 |
| Sulfonylureas | 346 (12.8) | 662 (24.6) | 269 (10.0) | 78 (2.9) | 12 (0.4) | <7 | <7 | <7 |
| SGLT-2 inhibitors | 70 (2.6) | 550 (20.4) | 445 (16.5) | 295 (10.9) | 108 (4.0) | 17 (0.6) | <7 | <7 |
| DPP-4 inhibitors | 104 (3.9) | 543 (20.1) | 518 (19.2) | 165 (6.1) | 27 (1.0) | <7 | <7 | <7 |
| Thiazolidinediones | 31 (1.1) | 169 (6.3) | 127 (4.7) | 36 (1.3) | 9 (0.3) | <7 | <7 | <7 |
| Insulin | <7 | 107 (4.0) | 124 (4.6) | 134 (5.0) | 46 (1.7) | 13 (0.5) | <7 | <7 |
| Other glucose-lowering drugs | <7 | 11 (0.4) | <7 | <7 | <7 | <7 | <7 | <7 |
| Abbreviations: GLP-1 glucagon-like peptide-1, SGLT-2 sodium-glucose co-transporter 2, DPP-4 dipeptidyl peptidase-4. *First-line antidiabetic treatment was defined as the first non-insulin glucose-lowering therapy prescribed, while second-line to eighth-line treatments were defined as the initiation of any new glucose-lowering therapy, including insulin, not used in a previous treatment-line. †Most recent measurement recorded within the year before or at GLP-1 receptor agonist initiation. | | | | | | | | |

| **Table S14. Characteristics of individuals initiating glucagon-like peptide-1 (GLP-1) receptor agonist therapy between 01 January 2018 and 30 June 2023, stratified by sex assigned at birth.** | | | |
| --- | --- | --- | --- |
| **Characteristics** | **Initiation post-2018** | **Female** | **Male** |
| Individuals included, n (%) | 11263 (100.0) | 5034 (44.7) | 6229 (55.3) |
| Age at initiation, years, median (IQR) | 60.8 [53.1, 68.6] | 60.0 [52.6, 68.3] | 61.1 [53.7, 68.8] |
| Female sex assigned at birth, n (%) | 5034 (44.7) | 5034 (100.0) | <7 |
| Type 2 diabetes duration, years, median (IQR) | 6.7 [3.2, 11.5] | 6.2 [2.9, 10.9] | 7.1 [3.5, 11.9] |
| **GLP-1 receptor agonist agent initiated, n (%)** | | | |
| Semaglutide | 5084 (45.1) | 2203 (43.8) | 2881 (46.3) |
| Dulaglutide | 4662 (41.4) | 2098 (41.7) | 2564 (41.2) |
| Liraglutide | 1234 (11.0) | 602 (12.0) | 632 (10.1) |
| Exenatide | 241 (2.1) | 108 (2.1) | 133 (2.1) |
| Lixisenatide | 42 (0.4) | 23 (0.5) | 19 (0.3) |
| **Previous use of glucose-lowering therapies*, n (%)** | | | |
| Metformin | 10932 (97.1) | 4849 (96.3) | 6083 (97.7) |
| Sulfonylureas | 6286 (55.8) | 2728 (54.2) | 3558 (57.1) |
| SGLT-2 inhibitors | 6827 (60.6) | 2921 (58.0) | 3906 (62.7) |
| DPP-4 inhibitors | 6456 (57.3) | 2797 (55.6) | 3659 (58.7) |
| Thiazolidinediones | 1725 (15.3) | 672 (13.3) | 1053 (16.9) |
| Insulin | 1448 (12.9) | 700 (13.9) | 748 (12.0) |
| Other glucose-lowering drugs | 132 (1.2) | 47 (0.9) | 85 (1.4) |
| **Concurrent use of glucose-lowering therapies†, n (%)** | | | |
| Metformin | 9535 (84.7) | 4033 (80.1) | 5502 (88.3) |
| Sulfonylureas | 4349 (38.6) | 1854 (36.8) | 2495 (40.1) |
| SGLT-2 inhibitors | 5723 (50.8) | 2357 (46.8) | 3366 (54.0) |
| DPP-4 inhibitors | 4902 (43.5) | 2098 (41.7) | 2804 (45.0) |
| Thiazolidinediones | 500 (4.4) | 166 (3.3) | 334 (5.4) |
| Insulin | 1304 (11.6) | 628 (12.5) | 676 (10.9) |
| Other glucose-lowering drugs | 27 (0.2) | 7 (0.1) | 20 (0.3) |
| **Comorbidities‡, n (%)** | | | |
| Cardiovascular disease | 2103 (18.7) | 716 (14.2) | 1387 (22.3) |
| Hypertension | 7174 (63.7) | 3143 (62.4) | 4031 (64.7) |
| Dyslipidemia | 2189 (19.4) | 877 (17.4) | 1312 (21.1) |
| Depression | 4620 (41.0) | 2626 (52.2) | 1994 (32.0) |
| Asthma | 2540 (22.6) | 1439 (28.6) | 1101 (17.7) |
| Chronic obstructive pulmonary disease | 2891 (25.7) | 1535 (30.5) | 1356 (21.8) |
| Chronic kidney disease | 1774 (15.8) | 845 (16.8) | 929 (14.9) |
| **Laboratory and vital sign measurements§** | | | |
| BMI, kg/m^2^, median (IQR) | 34.0 [30.5, 38.0] | 34.9 [31.2, 39.0] | 33.2 [30.1, 37.2] |
| BMI ≥35, n (%) | 3654 (32.4) | 1789 (35.5) | 1865 (29.9) |
| BMI missing, n (%) | 2696 (23.9) | 1384 (27.5) | 1312 (21.1) |
| eGFR, ml/min per 1.72m^2^, median (IQR) | 98.20 [82.00, 107.70] | 103.3 [92.9, 111.8] | 93.4 [74.5, 103.0] |
| eGFR <60, n (%) | 1075 (9.5) | 301 (6.0) | 774 (12.4) |
| Missing eGFR, n (%) | 334 (3.0) | 135 (2.7) | 199 (3.2) |
| HbA1c, %, median (IQR) | 8.19 [7.64, 8.55] | 8.10 [7.55, 8.55] | 8.19 [7.64, 8.65] |
| **Line of glucose-lowering therapy at GLP-1 receptor agonist initiation, n (%)** | | | |
| First-line | 160 (1.4) | 95 (1.9) | 65 (1.0) |
| Second-line | 1463 (13.0) | 716 (14.2) | 747 (12.0) |
| Third-line | 2713 (24.1) | 1193 (23.7) | 1520 (24.4) |
| Fourth-line | 3475 (30.8) | 1560 (31.0) | 1915 (30.7) |
| Fifth-line | 2519 (22.4) | 1074 (21.3) | 1445 (23.2) |
| Sixth to eighth-line | 933 (8.3) | 396 (7.9) | 537 (8.6) |
| Abbreviations: GLP-1 glucagon-like peptide-1, SGLT-2 sodium-glucose co-transporter 2, DPP-4 dipeptidyl peptidase-4, HbA1c glycated hemoglobin, BMI body-mass index, eGFR estimated glomerular filtration rate.  *Defined as having at least one prescription for a glucose-lowering therapy specified in Supplementary Table S2 before GLP-1 receptor agonist initiation. †Defined as having at least one prescription for a glucose-lowering therapy specified in Supplementary Table S2 within the 180 days before or at GLP-1 receptor agonist initiation. ‡Diagnosis recorded before or at GLP-1 receptor agonist initiation. §Most recent measurement recorded within the year before or at GLP-1 receptor agonist initiation. | | | |

| **Table S15. Glucose-lowering therapies (GLT)* used during the one year before and after glucagon-like peptide-1 (GLP-1) receptor agonist initiation in 2018 or later, stratified by sex assigned at birth.** | | | | |
| --- | --- | --- | --- | --- |
| **Use of GLT* within time intervals (days) respective to GLP-1 receptor agonist therapy initiation (day 0)** | **-361, -181** | **-180, 0** | **1, 181** | **182, 362** |
| **GLP-1 receptor agonist therapy initiation between 01-Jan-2018 and 30-Jun-2023: Females** | | | | |
| Females, n (%) | 5034 (100.0) | 5034 (100.0) | 3076 (100.0) | 2154 (100.0) |
| Metformin, n (%) | 3847 (76.4) | 4033 (80.1) | 2375 (77.2) | 1634 (75.9) |
| Sulfonylureas, n (%) | 1737 (34.5) | 1854 (36.8) | 976 (31.7) | 639 (29.7) |
| SGLT-2 inhibitors, n (%) | 2068 (41.1) | 2357 (46.8) | 1092 (35.5) | 768 (35.7) |
| DPP-4 inhibitors, n (%) | 1977 (39.3) | 2098 (41.7) | 323 (10.5) | 119 (5.5) |
| Thiazolidinediones, n (%) | 163 (3.2) | 166 (3.3) | 48 (1.6) | 31 (1.4) |
| Other GLT, n (%) | 8 (0.2) | 7 (0.1) | <7 | <7 |
| Insulin, n (%) | 519 (10.3) | 628 (12.5) | 361 (11.7) | 269 (12.5) |
| Number of co-prescribed GLT |  | | | |
| GLT=0, n (%) | 399 (7.9) | 126 (2.5) | 214 (7.0) | 176 (8.2) |
| GLT=1, n (%) | 1070 (21.3) | 1042 (20.7) | 1079 (35.1) | 791 (36.7) |
| GLT=2, n (%) | 1739 (34.5) | 1838 (36.5) | 1295 (42.1) | 919 (42.7) |
| GLT=3, n (%) | 1544 (30.7) | 1699 (33.8) | 446 (14.5) | 241 (11.2) |
| GLT≥3, n (%) | 1826 (36.3) | 2028 (40.3) | 488 (15.9) | 268 (12.4) |
| GLT≥4, n (%) | 282 (5.6) | 329 (6.5) | 42 (1.4) | 27 (1.3) |
| **GLP-1 receptor agonist therapy initiation between 01-Jan-2018 and 30-Jun-2023: Males** | | | | |
| Males, n (%) | 6229 (100.0) | 6229 (100.0) | 3759 (100.0) | 2641 (100.0) |
| Metformin, n (%) | 5299 (85.1) | 5502 (88.3) | 3303 (87.9) | 2305 (87.3) |
| Sulfonylureas, n (%) | 2393 (38.4) | 2495 (40.1) | 1299 (34.6) | 882 (33.4) |
| SGLT-2 inhibitors, n (%) | 2981 (47.9) | 3366 (54.0) | 1654 (44.0) | 1176 (44.5) |
| DPP-4 inhibitors, n (%) | 2664 (42.8) | 2804 (45.0) | 411 (10.9) | 150 (5.7) |
| Thiazolidinediones, n (%) | 343 (5.5) | 334 (5.4) | 136 (3.6) | 93 (3.5) |
| Other GLT, n (%) | 20 (0.3) | 20 (0.3) | 11 (0.3) | 7 (0.3) |
| Insulin, n (%) | 571 (9.2) | 676 (10.9) | 382 (10.2) | 269 (10.2) |
| Number of co-prescribed GLT |  | | | |
| GLT=0, n (%) | 346 (5.6) | 78 (1.3) | 124 (3.3) | 90 (3.4) |
| GLT=1, n (%) | 998 (16.0) | 912 (14.6) | 1028 (27.3) | 775 (29.3) |
| GLT=2, n (%) | 2012 (32.3) | 2128 (34.2) | 1730 (46.0) | 1255 (47.5) |
| GLT=3, n (%) | 2263 (36.3) | 2439 (39.2) | 804 (21.4) | 488 (18.5) |
| GLT≥3, n (%) | 2873 (46.1) | 3111 (49.9) | 877 (23.3) | 521 (19.7) |
| GLT≥4, n (%) | 610 (9.8) | 672 (10.8) | 73 (1.9) | 33 (1.2) |
| Abbreviations: GLT glucose-lowering therapies, GLP-1 glucagon-like peptide-1, CVD cardiovascular disease, SGLT-2 sodium-glucose co-transporter 2, DPP-4 dipeptidyl peptidase-4. *Defined as having at least one prescription for GLT specified in Supplementary Table S2 within the specified time interval, while adhering to GLP-1 receptor agonist therapy upon initiation. | | | | |

| **Table S16. Glucose-lowering therapies from the first prescription up to glucagon-like peptide-1 (GLP-1) receptor agonist initiation between 01 January 2018 and 30 June 2023, stratified by treatment line* and sex assigned at birth.** | | | | | | | | |
| --- | --- | --- | --- | --- | --- | --- | --- | --- |
| **Glucose-lowering therapies among females, n (%)** | **First-line** | **Second-line** | **Third-line** | **Fourth-line** | **Fifth-line** | **Sixth-line** | **Seventh-line** | **Eighth-line** |
| Individuals | 5034 (100.0) | 4939 (98.1) | 4223 (83.9) | 3030 (60.2) | 1470 (29.2) | 396 (7.9) | 52 (1.0) | <7 |
| GLP-1 receptor agonist | 95 (1.9) | 716 (14.2) | 1193 (23.7) | 1560 (31.0) | 1074 (21.3) | 344 (6.8) | 49 (1.0) | <7 |
| Metformin | 4585 (91.1) | 254 (5.0) | 33 (0.7) | <7 | <7 | <7 | <7 | <7 |
| Sulfonylureas | 575 (11.4) | 1346 (26.7) | 584 (11.6) | 207 (4.1) | 23 (0.5) | <7 | <7 | <7 |
| SGLT-2 inhibitors | 132 (2.6) | 1068 (21.2) | 947 (18.8) | 604 (12.0) | 183 (3.6) | 24 (0.5) | <7 | <7 |
| DPP-4 inhibitors | 172 (3.4) | 1138 (22.6) | 1078 (21.4) | 358 (7.1) | 47 (0.9) | <7 | <7 | <7 |
| Thiazolidinediones | 51 (1.0) | 292 (5.8) | 214 (4.3) | 76 (1.5) | 39 (0.8) | <7 | <7 | <7 |
| Insulin | <7 | 154 (3.1) | 195 (3.9) | 237 (4.7) | 105 (2.1) | 19 (0.4) | <7 | <7 |
| Other glucose-lowering drugs | 9 (0.2) | 15 (0.3) | 13 (0.3) | <7 | <7 | <7 | <7 | <7 |
| **Glucose-lowering therapies among males, n (%)** | **First-line** | **Second-line** | **Third-line** | **Fourth-line** | **Fifth-line** | **Sixth-line** | **Seventh-line** | **Eighth-line** |
| Individuals | 6229 (100.0) | 6164 (99.0) | 5417 (87.0) | 3897 (62.6) | 1982 (31.8) | 537 (8.6) | 75 (1.2) | <7 |
| GLP-1 receptor agonist | 65 (1.0) | 747 (12.0) | 1520 (24.4) | 1915 (30.7) | 1445 (23.2) | 462 (7.4) | 71 (1.1) | <7 |
| Metformin | 5650 (90.7) | 412 (6.6) | 38 (0.6) | 11 (0.2) | <7 | <7 | <7 | <7 |
| Sulfonylureas | 904 (14.5) | 1692 (27.2) | 723 (11.6) | 235 (3.8) | 34 (0.5) | <7 | <7 | <7 |
| SGLT-2 inhibitors | 175 (2.8) | 1304 (20.9) | 1196 (19.2) | 964 (15.5) | 287 (4.6) | 40 (0.6) | <7 | <7 |
| DPP-4 inhibitors | 256 (4.1) | 1444 (23.2) | 1430 (23.0) | 468 (7.5) | 61 (1.0) | <7 | <7 | <7 |
| Thiazolidinediones | 115 (1.8) | 458 (7.4) | 335 (5.4) | 106 (1.7) | 37 (0.6) | <7 | <7 | <7 |
| Insulin | <7 | 180 (2.9) | 199 (3.2) | 232 (3.7) | 119 (1.9) | 34 (0.5) | <7 | <7 |
| Other glucose-lowering drugs | 16 (0.3) | 25 (0.4) | 26 (0.4) | 10 (0.2) | 8 (0.1) | <7 | <7 | <7 |
| Abbreviations: GLP-1 glucagon-like peptide-1, SGLT-2 sodium-glucose co-transporter 2, DPP-4 dipeptidyl peptidase-4. *First-line treatment was defined as the first non-insulin glucose-lowering therapy prescribed, while second-line to eighth-line treatments were defined as the initiation of any new glucose-lowering therapy, including insulin, not used in a previous treatment-line. | | | | | | | | |

SUPPLEMENTARY FIGURES

| **** |
| --- |
| **Figure S1. Cohort construction of individuals initiating glucagon-like peptide-1 (GLP-1) receptor agonist therapy using the IQVIA Medical Research Data (IMRD) incorporating data from THIN, A Cegedim Database.** |

| **c** |
| --- |
| **Figure S2. Sankey diagram showing the trajectories of glucose-lowering therapies from the first prescription to the initiation of glucagon-like peptide-1 (GLP-1) receptor agonist therapy among individuals initiating GLP-1 receptor agonist therapy between 01-Jan-2007 to 30-Jun-2023, stratified by treatment line*.** |
| Abbreviations: GLP-1RA glucagon-like peptide-1 receptor agonist, SGLT-2 sodium-glucose co-transporter 2, DPP-4 dipeptidyl peptidase-4. *First-line treatment was defined as the first non-insulin glucose-lowering therapy prescribed, while second-line to eighth-line treatments were defined as the initiation of any new glucose-lowering therapy, including insulin, not used in a previous treatment line. For clarity, trajectories are displayed up to the fifth treatment line; glucose-lowering therapies shown in the fifth line represent individuals who initiated a GLP-1RA at the sixth-line or later. The percentage of individuals initiating a specific a glucose-lowering therapy for a given treatment line are displayed in the columns (top three percentages per column are shown, while the numbers across all treatment lines (i.e., first to eighth) can be found in Supplementary Table S6 and the diagrams provide a visual summary of treatment sequencing across lines). |

|  |
| --- |
| **Figure S3. Sankey diagram showing the trajectories of glucose-lowering therapies from the first prescription to the initiation of glucagon-like peptide-1 (GLP-1) receptor agonist therapy among individuals initiating GLP-1 receptor agonist therapy between 01-Jan-2018 to 30-Jun-2023, stratified by treatment line* and cardiovascular disease (CVD) history at GLP-1 receptor agonist initiation (A, B).** |
| Abbreviations: GLP-1RA glucagon-like peptide-1 receptor agonist, SGLT-2 sodium-glucose co-transporter 2, DPP-4 dipeptidyl peptidase-4. *First-line treatment was defined as the first non-insulin glucose-lowering therapy prescribed, while second-line to eighth-line treatments were defined as the initiation of any new glucose-lowering therapy, including insulin, not used in a previous treatment line. For visual clarity, trajectories are displayed up to the fifth treatment line; glucose-lowering therapies shown in the fifth line represent individuals who initiated a GLP-1RA at the sixth-line or later. The percentage of individuals initiating a specific a glucose-lowering therapy for a given treatment line are displayed in the columns (top three percentages per column are shown, while the numbers across all treatment lines can be found in Supplementary Table S10 and the diagrams provide a visual summary of treatment sequencing across lines). |

| **c** |
| --- |
| **Figure S4. Sankey diagram showing the trajectories of glucose-lowering therapies from the first prescription to the initiation of glucagon-like peptide-1 (GLP-1) receptor agonist therapy among individuals initiating GLP-1 receptor agonist therapy between 01-Jan-2018 to 30-Jun-2023, stratified by treatment line* and body-mass index (BMI)† at GLP-1 receptor agonist initiation (A, B).** |
| Abbreviations: GLP-1RA glucagon-like peptide-1 receptor agonist, SGLT-2 sodium-glucose co-transporter 2, DPP-4 dipeptidyl peptidase-4. *First-line treatment was defined as the first non-insulin glucose-lowering therapy prescribed, while second-line to eighth-line treatments were defined as the initiation of any new glucose-lowering therapy, including insulin, not used in a previous treatment line. For visual clarity, trajectories are displayed up to the fifth treatment line; glucose-lowering therapies shown in the fifth line represent individuals who initiated a GLP-1RA at the sixth-line or later. The percentage of individuals initiating a specific a glucose-lowering therapy for a given treatment line are displayed in the columns (top three percentages per column are shown, while the numbers across all treatment lines can be found in Supplementary Table S13 and the diagrams provide a visual summary of treatment sequencing across lines). †Most recent measurement recorded within the year before or at GLP-1 receptor agonist initiation. |

| **c** |
| --- |
| **Figure S5. Sankey diagram showing the trajectories of glucose-lowering therapies from the first prescription to the initiation of glucagon-like peptide-1 (GLP-1) receptor agonist therapy among individuals initiating GLP-1 receptor agonist therapy between 01-Jan-2018 to 30-Jun-2023, stratified by treatment line* and sex assigned at birth (A, B).** |
| Abbreviations: GLP-1RA glucagon-like peptide-1 receptor agonist, SGLT-2 sodium-glucose co-transporter 2, DPP-4 dipeptidyl peptidase-4. *First-line antidiabetic treatment was defined as the first non-insulin glucose-lowering therapy prescribed, while second-line to eighth-line treatments were defined as the initiation of any new glucose-lowering therapy, including insulin, not used in a previous treatment line. For visual clarity, trajectories are displayed up to the fifth treatment line; glucose-lowering therapies shown in the fifth line represent individuals who initiated a GLP-1RA at the sixth-line or later. The percentage of individuals initiating a specific a glucose-lowering therapy for a given treatment line are displayed in the columns (top three percentages per column are shown, while the numbers across all treatment lines can be found in Supplementary Table S16 and the diagrams provide a visual summary of treatment sequencing across lines). |
